# 33rd Annual Meeting of the Society for Light Treatment and Biological Rhythms (SLTBR), 23–25 June 2022, Manchester, UK

**DOI:** 10.3390/clockssleep4030035

**Published:** 2022-09-02

**Authors:** Marijke C. Gordijn

**Affiliations:** 1 Chrono@Work, Friesestraatweg 213, 9743 AD Groningen, The Netherlands; marijke.gordijn@chronoatwork.com; 2 Chronobiology Unit, Groningen Institute for Evolutionary Life Sciences, University of Groningen, 9747 AG Groningen, The Netherlands

## 1. Introduction

The 33rd annual meeting of the Society for Light Treatment and Biological Rhythms was held in Manchester in June 2022. The meeting provided high-quality scientific contributions in the fundamental, translational and clinical fields with respect to the effects of light on the organism and the chronobiology of psychiatric as well as other medical disorders. Renowned international speakers from all over the world provided comprehensive updates and discussed novel scientific topics varying from the effects of light on circadian rhythms, on female reproductive cycle, on sleep, as well as on cognition and performance. Attention was given to the application of light, for instance, in occupational settings as well as for clinical purposes such as light treatment in neurodegenerative disorders, following traumatic brain injury, as well as for perinatal depression. A special CPD course was dedicated to the role and everyday usage of melatonin and melatonin agonists in circadian rhythm sleep wake disorders and neuropsychiatric disorders. Free oral contributions covered various cutting-edge technologies such as fMRI studies and metameric displays to unravel the mechanisms behind non-visual effects of light. Young scientists met with more experienced scientists during the young investigator symposium and the poster session. Last but not least, after a break of two years because of the COVID-19 pandemic, friends and colleagues met each other again, made new friends, engaged in discussions and went home with new inspirations.

## 2. Conference Abstracts

### 2.1. Detecting Individual-Specific Changes in Circadian Rest-Activity Rhythm and Sleep in Individuals Tapering Their Antidepressant Medication

Olga Minaeva ^1^, Evelien Schat ^2^, Eva Ceulemans ^2^, Yoram Kunkels ^1^, Arnout Smit ^1^, Marieke Wichers ^1^, Sanne Booij ^1^ and Harriëtte Riese ^1^

^1^  Interdisciplinary Center Psychopathology and Emotion regulation, Department of Psychiatry, University Medical Center Groningen, University of Groningen
^2^  KU Leuven, Faculty of Psychology and Educational Sciences

**Abstract: Background**: Depressed individuals tend to have more disrupted sleep and circadian rest-activity rhythms (RARs) compared to non-depressed individuals. However, whether this disruption is temporally linked to a transition in depressive symptoms is unclear. Furthermore, most of the prior research findings come from group-level studies. Hence, it remains uncertain whether these associations replicate at the within-person level. Therefore, it is clinically relevant to investigate at the individual level whether changes in depressive symptoms are related to changes in circadian rhythm and sleep variables. In the current replicated single-subject study, we aimed (1) to identify changes in circadian rhythm elements (RAR, physical activity, and sleep) in proximity to a transition in depressive symptoms, (2) whether changes are more frequent in individuals with a transition, compared to those without, and (3) whether there are individual differences in the direction of change of circadian rhythm variables. **Methods**: Four months of actigraphy data of remitted individuals who tapered their antidepressant medication during the study period and were therefore at high risk of developing depressive symptoms were used. In total, 12 individuals with and 14 without a transition in depressive symptoms during the study period were included. RAR, physical activity, and sleep variables were calculated as predictors from the actigraphy data. The outcome was the timing of transition in depressive symptoms based on the weekly SCL-90 depression subscale scores, evaluation interviews, and self-reported experience of recurrence of depressive symptoms. Kernel Change Point analyses were used to detect change points (CPs) and CP timing in circadian rhythm variables for each individual separately. **Results**: We identified CPs at the individual level for most participants with transitions (69%, 9 out of 13). This means that most of the participants with transitions exhibited changes in their RAR, PA, and sleep around the transition time large enough to be detected by the change point analysis. Overall, CPs were detected less frequently in individuals without a transition, with 0.64 CPs per individual on average, compared to those with a transition in depressive symptoms with 1 CP per individual on average. CPs were detected both preceding and following an increase in depressive symptoms. The direction of changes was found to vary between individuals. However, some variables showed a more consistent pattern in direction than others: increased time in bed and IV (intradaily variability) and decreased MESOR (the rhythm-adjusted mean) and amplitude. **Conclusions**: Circadian rhythm variables provide potentially clinically relevant information, although their patterns around transitions are highly person-specific. Considering the timing of identified CPs, we can hypothesize that for different individuals, sleep and circadian rhythm can play a different role in the development of depression, with likely different mechanisms at play. To use this finding in mental health care, more empirical single-subject research is needed on individual differences in the amount and direction of changes. 

**Keywords**: circadian rhythm; sleep; depression; individual models; transition

**Funding:** This study was supported by the European Research Council (ERC) under the European Union’s Horizon 2020 research and innovative programme (ERC-CoG-2015; No. 681466 to M. Wichers).

### 2.2. Novel Correlates of Immediate Response to Chronotherapeutics: Normalization of Prefrontal Glutamate, Inflammation, White Matter Microstructure, and Brain Functional Connectivity

Francesco Benedetti ^1,2^

^1^  University Vita-Salute San Raffaele, Milno
^2^  Scientific Institute IRCCS Ospedale San Raffaele, Milano

**Abstract: Background**: One-third of patients with bipolar disorder (BD) attempt suicide. Depression in BD associates with drug-resistance, hence the urgent need of alternative rapid treatment options. Total sleep deprivation (SD) and light therapy (LT) prompt a rapid and stable antidepressant response in BD, and can provide hints about new therapeutic targets. Previous studies associated BD and suicidal ideation with disrupted white matter (WM) microstructure, reduced grey matter (GM) volumes, altered immune-inflammatory setpoints, and abnormal functional cortico-limbic connectivity during emotional processing. **Methods**: In recent years, we studied 204 (121 females, 83 M) depressed inpatients with BD, treated with repeated SD + LT (three cycles in one week), to identify new biomarkers of efficacy. Subsamples were studied with multimodal MRI imaging before/after treatment: diffusion tensor imaging (DTI) for white matter (WM) microstructure, voxel-based morphometry (VBM) and cortical thickness for grey matter (GM) structure, BOLD neural activation and functional connectivity parameter changes in response to a cortico-limbic activating task, and single-proton magnetic resonance spectroscopy (1H-MRS) in the dorsolateral prefrontal cortex (DLPFC). Peripheral levels of IL-1β and its receptor antagonist (IL-1β:IL1ra) were measured as markers of inflammation. **Results**: After treatment, 137 patients (67.2%) achieved response (HDRS score < 8). All patients showed an immediate decrease of suicidal ideation, soon after the first SD cycle and irrespective of final response. This effect was significant in non-responders, too. Multimodal imaging detected several correlates of treatment effects. 1H-MRS showed both, Glutamate and the composite measure of Glutamate+Glutamine (Glx) significantly decreased after treatment, the latter marking inflammation and correlating with WM microstructure. For DTI, TBSS showed an increase of fractional anisotropy (FA) and a decrease of mean diffusivity (MD), spread in the WM skeleton, which was proportional to clinical response (Delta HDRS). Peak effects of treatment were observed in corpus callosum, the hippocampal part of the cingulate gyrus, uncinatus, fornix, sagittal striatum and internal capsule. For BOLD fMRI, significant interactions between task and response to chronotherapeutics were found for connectivity between left amygdala and several structures, including hippocampus, insula, supramarginal gyrus and occipital cortex. VBM showed increased GM volumes after treatment in BA 39–40 and right caudate, with no significant changes in cortical thickness. Treatment significantly decreased IL-1β:IL1ra, the effect being proportional to baseline levels and normalizing higher values; patients with higher baseline levels showed the highest decrease in IL-1β:IL-1ra, proportional to antidepressant response. **Conclusions**: Adding up to the known enhancement of monoaminergic neurotransmission, chronotherapeutics can modulate glutamatergic neurotransmission, foster a neurotrophic/antinflammatory milieu, prompt cortical neuroplasticity, counteract disruption of white matter microstructure and water homeostasis and improve top-down cortico-limbic effective connectivity. These mechanisms are likely to contribute to its efficacy. 

### 2.3. How Spatial Patterns of Visual Scenes Change Rodents’ Behavioral States

Qian Huang, Robert Lucas and Riccardo Storchi

University of Manchester, Manchester, UK

Spatial patterns are an unavoidable feature of our visual experience, and convey useful information about the characteristics of an individual’s local environment. Thus, it is crucial to consider the impact of spatial patterns when studying animals’ response to illumination, especially when considering the long-term modulation for animal behaviors. The objective of my research is to determine how spatial patterns of visual scenes change rodents’ behavioral states. I have built a behavioral arena with two compartments where the spatial distribution of light could be quantitatively controlled in each compartment. The experimental compartment contains spatially patterned light across elevations (simulating the appearance of local shade), while the control compartment refers to a relatively uniform light (simulating an open field). Mice could pass freely between two chambers and their voluntary locomotion could be recorded and analyzed. In behavioral trials, mice preferred to spend more time in the experimental compartment where the light was spatially distributed. The difference in light intensity between two chambers from −20° to 20° could impact mice preference to the greatest degree. Thus, we concluded that mice could show different exploratory preferences when facing visual environments with various spatial patterns in light intensity across elevations. The difference in light intensity in a specific range might play a dominant role in mice’s exploratory preference. 

**Keywords**: spatial patterns; behavioral states; exploratory preference; elevation range

**Funding:** The project has received funding from the European Union’s Horizon 2020 research and innovation programme under the Marie Skłodowska-Curie grant agreement No. 860613.

### 2.4. Salivary Cortisol Awakening Response as Predictor for Depression Severity

Else Refsgaard ^1^, Anne Vibeke Schmedes ^2^ and Klaus Martiny ^3^

^1^  Psychiatric Research Unit, Mental Health Centre North Zealand, Denmark
^2^  Department of Biochemistry and Immunology, Lillebaelt Hospital, Vejle, Denmark
^3^  New Interventions in Depression (NID) Group, Copenhagen Affective Disorder Research Center (CADIC), Mental Health Centre Copenhagen, Rigshospitalet, University of Copenhagen, Copenhagen, Denmark

**Abstract: Background**: The hypothalamic–pituitary–adrenal axis function in depression has for decades been investigated with a variety of methods. We investigated associations between the saliva cortisol awakening response (CAR) with endpoint depression scores and the baseline 24 h urine cortisol output with concurrent CAR measures. The purpose was to test any association between CAR measures and endpoint depression scores and between 24 h urine cortisol output and CAR measures. **Methods**: Patients with a major depressive episode were treated with duloxetine, delivered saliva samples at awakening and 15, 30, and 60 min post-awakening, and sampled urine for 24 h. Patients afterwards started a daily exercise program maintained for the 9-week period. Clinician-rated depression severity was weekly assessed with the Hamilton Depression Rating six-item subscale (HAM-D6). From the cortisol awakening response, the area under the curve was calculated with respect to the ground (AUCG) and with respect to the rise (AUCI) using saliva cortisol levels in a 1 h period after awakening. Associations between depression severity, AUCG, AUCI, exercise and 24 h cortisol output were performed in a general linear model. **Results**: The mean age of the 35 participants was 49.0 years (11.0) and with a mean baseline HAM-D6 score of 12.2 (2.3). In the statistical model investigating the association between HAM-D6 at week 9 as a dependent variable and AUCI, concurrent HAM-D6, gender, smoking and exercise volume as covariates, we found a significant effect of AUCI, concurrent HAM-D6, and exercise. The following statistics were found: AUCI (regression coefficient 0.008; F value = 9.1; *p* = 0.007), concurrent HAM-D6 (regression coefficient 0.70; F value = 8.0; *p* = 0.01), and exercise (regression coefficient −0.005; F value = 5.7; *p* = 0.03). The model had an R2 of 0.43. This association between HAM-D6 endpoint scores and the AUCI showed that higher AUCI values predicted higher HAM-D6 endpoint values. The association between HAM-D6 endpoint scores and the exercise level showed that a high exercise level was associated with lower HAM-D6 endpoint values. **Conclusions**: This is a little step toward finding easy-to-implement methods to predict depression outcomes and thus being able to allocate more resources to those patients that are predicted to have a less favorable outcome. It illustrates the use of circadian measures in clinical psychiatry.

**Keywords**: depression; cortisol awakening response; prediction; HPA-axis

### 2.5. Optimizing a Dynamic Lighting System in New Psychiatry Bispebjerg

Klaus Martiny ^1^, Carlo Volf ^1^, Paul Michael Petersen ^2^ and Torben Hansen ^3^

^1^  Mental health services in the Capital Region of Denmark, Brondby, Denmark
^2^  Department of Photonics Engineering, Technical University of Denmark, Kongens Lyngby, Denmark
^3^  Chromaviso A/S, Aarhus, Denmark

**Abstract: Background**: Light dosed with adequate intensity and temporal timing has been shown to have a positive therapeutic effect in the treatment of both seasonal and non-seasonal depression. Light typically has been delivered for shorter periods in the morning, through a lightbox with a fixed intensity and spectral distribution. The development of the Light Emitting Diode (LED) technology, though, has made it possible to replace standard electrical room lighting with dynamic LED systems that can change light intensity and spectral distribution throughout the 24 h day to impact circadian rhythms, sleep and mood. LEDs have markedly lower energy consumption. Therefore, many new buildings have LED systems, often with dynamic controlled lighting. However, research into the settings of these dynamic LED systems is sparse, and no consensus has yet been reached. Therefore, we need to develop and test dynamic LED settings in buildings with different functions, when it comes to usability and specific effect measurements. In New Psychiatry Bispebjerg (NPB), we have been given the opportunity to develop and test a dynamic LED system, as part of the Mental Health service in the Capital Region of Denmark. The purpose of the study is to develop and test a novel dynamic LED setting in New Psychiatry Bispebjerg that can both reduce energy consumption and strengthen the circadian regulation, sleep, and mood of patients in the psychiatric wards in the NPB. **Methods**: Patients will be cluster-randomized to either a part of the ward with a standard dynamic lighting system, as provided by the contractors, or to a part of the ward with a novel, adjusted dynamic lighting system. The adjusted system will use a blue-enriched morning light with higher intensity in combination with low evening levels of blue-less lighting. A separate study will investigate the effect of optimizing patient rooms according to geographical placement in the building and seasons to optimize the lighting in all rooms of the building, and to compensate for differences caused by the geographical orientation. **Results**: The study will start collecting data at the end of 2022. **Conclusions**: The results will qualify the use of dynamic LED lighting systems in psychiatric hospitals and will also be able to qualify the general use of dynamic lighting systems in the built environment, together with a reduction in energy requirements. The New Psychiatry Bispebjerg is expected to be inaugurated in late 2022. The study period is planned to end at the end of 2024.

**Keywords**: circadian rhythms, LED, hospital, dynamic lighting, sleep, medication

### 2.6. Lumos Smart Glasses and Its Effects of Light Therapy Duration on Mood, Sleep and Cognition

Lucas Tang ^1^, Zhao Pan ^1^ and Jonathan Oakman ^2^

^1^  Lumos Health Inc., University of Waterloo, Waterloo, ON, Canada
^2^  University of Waterloo, Waterloo, ON, Canada

**Abstract**: Light has a fundamental impact on human biology; it triggers photoreceptors at the back of the retina to induce changes in the brain, affecting neurotransmitter levels such as melatonin and cortisol. For those struggling with depression and sleep, medical professionals generally recommend the use of a light therapy lamp for 30 min a day and the patient can improve within a few weeks of use. However, due to the practical limitations of current light therapy devices such as lamps and head gear, 30 min of therapy is often hard to adhere to and longer durations of light therapy are often considered infeasible. On the other hand, while the intensity of a light box is brighter than average indoor conditions, it rarely exceeds the brightness of the natural outdoors. Research also suggests that spending time outdoors could be just as effective as light therapy in treating seasonal affective disorder. As such, it is possible that light therapy’s role is to make up for a lack of natural light exposure. In this paper, it is hypothesized that the optimal benefits for light therapy can be achieved by simulating light conditions from natural daylight, meaning long durations of bright light during the day. For this to happen, it is crucial to overcome the technological and design barriers to receiving light therapy. In light of overcoming this barrier, a pair of light therapy glasses using an innovative coating was engineered. The glasses use a patent pending coating that reflects 480 nm electrically generated blue light from the temples, meanwhile allowing other wavelengths from the user’s environment to pass through, enabling regular vision while receiving therapy. The usability of the glasses was validated in a 10-person pilot study in 2020 funded by the Idea Center at the University of Notre Dame. The users were instructed to use the glasses for at least 30 min a day over a period of 8 weeks. By the end of the study, the users on average wore the glasses for 35 min a day (116% adherence) and experienced improvements of 13.3 points on the Montgomery Asberg depression rating scale. Afterwards, feedback from users was collected to improve design, reducing weight and improving comfort. In addition, a proprietary ambient light sensor measuring environmental melanopic lux and user adherence sensors was then engineered onto the glasses powered by a Bluetooth low energy chip. Apps were developed on iOS and Android to collect sensor data and upload to the cloud. The sensors and app work together to enable a function that adjusts the light output of the glasses based on the user’s ambient light levels and time of day. Two hundred of the new generation glasses were produced for a double blind clinical trial set to begin in late 2021. The trial aims to encourage users to use light therapy for as long as possible during the day, and study the effect of light therapy duration and its correlation with sleep, mood, reaction time and memory in the general population.

### 2.7. Circadian Reinforcement Therapy in Combination with Electronic Self-Monitoring Facilitates a Safe Post-Discharge Period for Patients with Depression

Klaus Martiny, Anne Sofie Aggestrup, Signe Dunker Svendsen, Philip Løventoft and Lasse Nørregaard

Mental health services in the Capital Region of Denmark, Brondby, Denmark

**Abstract**: **Background**: The transition from inpatient to outpatient treatment, for patients with depression, is associated with an increased risk of relapse and readmission. Patients, when discharged, are sensitive to changes and stressors and often lack support to help them keep the structure obtained in the inpatient ward. Loss of structure can lead to instability in the circadian system, disrupting mood and sleep. Our previous pilot trial showed that patients drifted with 29 min in sleep onset and 48 min in sleep offset in the four weeks after discharge. Day-to-day mood- and sleep-timing was very unstable. **Methods**: This trial aimed to enforce zeitgeber strength after discharge to stabilize mood and sleep. We developed a new concept ‘Circadian Reinforcement Therapy (CRT)’ and tested it in a randomized controlled trial. CRT uses specialized psychoeducation on important zeitgebers—light, exercise, diet and social contact—delivered before discharge and during follow-up. Patients entered mood, sleep and activity data into a closed-loop electronic system with trigger points for calls from clinicians in a four-week period. In addition, patients were called weekly to discuss their entered data and suggested how to correct rhythms and zeitgebers. The control group only entered data. **Results**: In all, 103 participants were included from September 2016 till November 2020. We had some difficulty recruiting participants before discharge and some participants found it difficult to take in new knowledge and learn new skills during this period. Readmission rates were low with 5.9% (3/51) in CRT and 9.6% (5/52) in the standard group. In a general linear model, we found a significantly higher reduction in depression (Hamilton scale) scores in the CRT group (*p* = 0.04) modified by the time of entry into the study (*p* = 0.005). The earlier, in relation to discharge, the patients were included in the study, the larger the separation seen between groups. Sleep onset was significantly earlier in the CRT group (*p* = 0.04) and sleep duration was longer (*p* = 0.01). The day-to-day instability of evening mood, sleep offset and sleep quality was significantly lower in the CRT group (all *p* < 0.001). **Conclusions**: CRT is a possible candidate for a new treatment method to stabilize sleep and improve mood in depressed patients. CRT should be tailored to be easier to use for depressed patients. The results highlight the importance of the circadian system in depression and are an example of yet another chronotherapeutic treatment method.

**Keywords**: Circadian rhythms; depression; sleep; zeitgebers; Circadian Reinforcement Therapy.

### 2.8. Clinical Outcomes of Light Therapy in Hospitalized Patients—A Systematic Review

Filippa Lindskov, Helle Iversen and Anders West

University Hospital of Copenhagen, Rigshospitalet, Copenhagen, Denmark

**Abstract: Background**: Light therapy and the effects on biological function have been known and investigated for decades. Light therapy is used to compensate for the lack of exposure to sunlight, which is thought to be linked to major depressive disorder with seasonal patterns. It is applied as sessions with bright light mimicking natural sunlight. Lack of bright light during daytime is not the only factor in maintaining the circadian rhythm; lack of exposure to bright light at night is also important. A new modality called naturalistic light shows promise, mimicking daylight by dynamically changing intensity and wavelengths throughout the day. Evidence of clinical effects, besides bright light effects on depression, is still limited, especially in hospital populations, and the present review aims to extract results of the effect of any optical light intervention on hospitalized patients. **Methods**: Through a database search, 29 trials were included, of which 8 trials used a variation of naturalistic light. Trials were heterogeneous regarding designs, populations, interventions, methods and outcomes. **Results**: In 14 out of 17 studies investigating sleep duration, quality and circadian alignment, along with decreased fatigue and improved mood in daytime, light therapy had a significant effect. Circadian rhythm and rhythmicity were affected as well. The effect on mood and cognition was inconsistent across studies. Trials showed more significant outcomes when conducted in non-intensive care units and with duration >5 days. Lux was reported in and compared across 24 studies and did not appear to be correlated to outcome; rather the distribution of wavelengths should be considered when conducting trials in the future. Of the eight trials investigating naturalistic light, four trials had significant outcomes and three had adverse outcomes compared to one in the standard light regime. **Conclusion**: The overall effect of light therapy is beneficial, but evidence for the effect of naturalistic light is still insufficient to warrant recommendation before other modalities. Future research in this area should be conducted in facilities where naturalistic light is installed, with a focus on the spectral distribution. 

**Keywords**: circadian; light; hospital; in-patient; naturalistic; review

### 2.9. Dim Light at Night during Pregnancy Affects the Rhythmicity of Hormones and Biochemical Parameters of the Rat Pups in Early Postnatal Ontogenesis

Zuzana Dzirbíková, Katarína Stebelová, Katarína Kováčová, Lucia Olexová and Michal Zeman

Department of Animal Physiology and Ethology, Faculty of Natural Sciences, Comenius University in Bratislava, Bratislava, Slovakia

**Abstract: Background**: Dim light at night (DLAN) is considered one of the most prevalent environmental pollutants in developed countries. DLAN can impact circadian rhythmicity of the endocrine system and metabolism with subsequent implications on health. In this perspective, DLAN can affect not only adults but also progeny if the mother is exposed to light at night during pregnancy. Our study aimed to assess the hormonal and biochemical parameters in pups of different postnatal ages whose mothers were exposed to DLAN during pregnancy. **Methods**: The experiment was performed with Wistar rats. Control dams (CTRL, n = 9) were housed on light:dark regime 12:12 (L = 500 lx, D = 0 lx) while dim light-exposed dams (DLAN, n =11) were housed on 12:12 light:dim light (L = 500 lx, dim light < 2 lx) during the whole pregnancy. On the day of the pup’s birth (P0), DLAN mothers with their pups were returned to LD with total dark. Animals were sacrificed at postnatal day 3 (P3), P10 and P20 in six points every 4 h (n = 6 pups/time point). Melatonin, corticosterone, triiodothyronine (T3) and thyroxine (T4) were measured by radioimmunoassay in the plasma and melatonin also in the pineal gland. Biochemical parameters (glucose, cholesterol, triglycerides) were measured with diagnostic kits. The 24 h rhythmicity was assessed by the cosinor analysis. **Results**: We found a significant rhythm in plasma melatonin in CTRL and DLAN pups of all age groups except for day P3 in the DLAN group. Plasma corticosterone was not rhythmic on P3 and P10 and the rhythm was identified on P20 in both groups. Importantly, in the DLAN group, the mesor and amplitude were decreased and acrophase was shifted as compared to CTRL. Thyroid hormones showed more variable daily profiles because T3 was rhythmic in CTRL and DLAN on P10, but not on P20 and T4 was rhythmic only in CTRL on P20. The main differences in biochemical parameters after DLAN exposure were found in glucose and cholesterol on P3 and in triglycerides on P10. These parameters were rhythmic in CTRL and lost their rhythmicity after DLAN. **Conclusions**: Exposure to DLAN during the pregnancy had an impact on the daily rhythmicity of measured hormones and biochemical parameters in rat pups during early ontogeny. Our results suggest that even a low level of artificial light at night during pregnancy can act as an endocrine disruptor, which can interfere with normal development. 

**Keywords**: dim light at night; melatonin; corticosterone; thyroid hormones; glucose; cholesterol; triglycerides; ontogeny

**Funding:** This study was supported by grants APVV-18-0174 and by the Operation Program of Integrated Infrastructure for the Project, Advancing University Capacity and Competence in Research, Development and Innovation, ITMS2014+: 313021X329, co-financed by the European Regional Development Fund.

### 2.10. The Impact of Short-Wavelength Reduction during the Day on Human Sleepiness and Reaction Time

Katarína Stebelová ^1^, Lucia Gracová ^1^, Katarína Kováčová ^1^, Zuzana Dzirbíková ^2^, Peter Hanuliak ^2^, Peter Hartman ^2^, Andrea Vargová ^2^ and Jozef Hraška ^2^

^1^  Department of Animal Physiology and Ethology, Faculty of Natural Sciences, Comenius University in Bratislava, Bratislava, Slovakia
^2^  Department of Building Structures, Faculty of Civil Engineering, Slovak University of Technology, Bratislava, Slovakia

**Abstract: Background**: Light has a substantial impact on the synchronization of biological rhythms. The positive effect especially of the blue light spectrum on work performance, reduced sleepiness and well-being during the day was described. Our study aimed to determine the effects of the short-wavelength light spectrum reduction during the working hours on working performance assessed by sleepiness, reaction time and response speed. **Methods**: Young healthy volunteers (23 volunteers—15 women, 8 men, average age 26 ± 1.5 years) participated in the study. The study was performed during the winter months (December 2020, November 2021–January 2022). The experiment took place in two identical rooms—a control room and a short-wavelength reduced room, where the participants spent time from 8:00–16:00 per day. During four control days, they were in the control room with natural lighting conditions and color temperature approx. 6000 K–6300 K. During five short-wavelength-reduced days, the windows and lamps in the short-wavelength-reduced room were covered with foil that filtered the light spectrum up to 500 nm (Orange 50 UV, KeetecFol, EU). The color temperature in that room was approx. 2700 K. During short-wavelength-reduced days participants used blue light filters on all the screens they used, even at home. Attentional tests—visual reaction time (VRT) and Go/NoGO test (GNG), and executive Eriksen flanker test (EFT)—were performed at 9:00, 11:00 and 15:00 each day. Participants completed a Karolinska sleepiness scale for determining subjective sleepiness at the same time points each day. **Results**: Short-wavelength light filtration significantly increased sleepiness in men compared to women, whereas under normal light conditions there were no differences in subjective sleepiness scores between men and women. The absence of short-wavelength light did not affect the reaction time when the men and women were evaluated together, regardless of the time when tests were performed and the type of test. Interestingly, when we evaluated reaction times according to sex there were differences due to the type of test. Men had significantly faster reaction times compared to women in the VRT test in both control and experimental conditions at all time points. In the GNG test, we found sex differences only at 9:00 in control conditions and at 11:00 in experimental conditions. There were no differences between men’s and women’s reaction times in EFT tests in any conditions. **Conclusions**: The absence of short-wavelength light during the daytime had a significant impact on the subjective sleepiness of men, but not women. We observed significant differences between the sexes in the reaction time in both lighting conditions, but these differences disappeared in tests dealing with the speed of reaction time and decision-making in both lighting conditions. Our results point to the importance of examining lighting conditions in the work environment also from a sex perspective.

**Keywords**: short-wavelength reduction; daylight exposure; reaction time; Karolinska sleepiness scale

**Funding:** The study was supported by grant APVV-18-0174.

### 2.11. Does the Reduction of Short-Wavelength Light during the Day Impact Human Sleep

Katarína Kováčová ^1^, Zuzana Dzirbíková ^1^, Peter Hanuliak ^2^, Peter Hartman ^2^, Andrea Vargová ^2^, Jozef Hraška ^2^ and Katarína Stebelová ^1^

^1^  Department of Animal Physiology and Ethology, Faculty of Natural Sciences, Comenius University in Bratislava, Bratislava, Slovakia
^2^  Department of Building Structures, Faculty of Civil Engineering, Slovak University of Technology, Bratislava, Slovakia

**Abstract: Background**: Light is the main synchronizing factor of the circadian system and provides the adaptation of organisms to rhythmic environmental conditions. Exposure to natural daylight ensures the proper functioning of the body, elevated vigilance during the day and better sleep at night. People spend most of the daytime in buildings under artificial lighting with reduced light intensity or a modified natural light spectrum. These conditions can have a negative impact on sleep and work performance. Our study aimed to find out in young healthy individuals (1) the effects of short-wavelength reduction during the daytime on the sleep parameters and (2) to determine the impact of evening intense light exposure depending on previous light history. **Methods**: In our study, 23 young healthy volunteers participated (14 women, 9 men, mean age 26 ± 1.5 years). The experiment took place in two identical rooms from 8:00 to 16:00 h. The light conditions in the rooms were monitored continuously by spectrophotometer and personal light exposure was monitored at the eye level by LightWatchers (Object-Tracker, AT). During four control days, participants spent the daytime in a room with natural lighting conditions (~6300 K). During five experimental days, participants spent the daytime in a room where the natural lighting was modified with foil (Orange 50 UV, KeetecFol, EU) that filtered light spectra up to 500 nm (~2700 K) and used blue light filters on their screens. In the evening under both conditions, participants used blue light filters two hours before sleep, except one night, when they were exposed to intense light before sleep. The activity and sleep parameters were monitored by MotionWatch 8 (CamNtech, Fenstanton, UK). **Results**: We observed significantly increased mobile minutes and mobile time during sleep after a stay in short-wavelength filtered light conditions in women. In men, we did not observe significant changes in sleep parameters. Under control conditions, 2 h evening light exposure before sleep caused a shortening of actual sleep, higher mobile time and lower sleep efficiency compared to nights with dim light evening exposure in women, not in men. Short-wavelength light-filtered environment caused after 2 h of light exposure before sleep changes many sleep parameters as shorter assumed sleep and actual sleep, lower sleep efficiency and a delayed mid-sleep time. Remarkably, these changes were observed only in women; the sleep of men was not affected. **Conclusions**: The reduced circadian potential in the daytime environment worsened the sleep parameters according to wrist actigraphy in women but not in men. The results suggest that exposure to light before bedtime influences sleep duration and quality depending on gender. 

**Keywords**: sleep; daylight; light history; short-wavelength light filtering; actigraphy

**Funding:** The study was supported by grant APVV-18-0174, UK/378/2022 and the Operation Program of Integrated Infrastructure for the project, Advancing University Capacity and Competence in Research, Development and Innovation, ITMS2014+: 313021X329, co-financed by the European Regional Development Fund.

### 2.12. The Use of Circadian Markers as Predictor of Response in the Treatment of Depression—A Systematic Review

Stella Druiven ^1^, Johanna Hovenkamp-Hermelink ^2^, Benno Haarman ^3^, Jeanine Kamphuis ^3^, Ybe Meesters ^3^, Harriëtte Riese ^1^ and Robert Schoevers ^1^

^1^  Department of Psychiatry, Research School of Behavioral and Cognitive Neurosciences (BCN), Interdisciplinary Center Psychopathology and Emotion regulation (ICPE), University Medical Center Groningen, University of Groningen, Groningen, The Netherlands
^2^  Department of Psychiatry, Interdisciplinary Center Psychopathology and Emotion Regulation (ICPE), University Medical Center Groningen, University of Groningen, Groningen, The Netherlands
^3^  Department of Psychiatry, Research School of Behavioral and Cognitive Neurosciences (BCN), University Medical Center Groningen, University of Groningen, Groningen, The Netherlands

**Abstract: Background**: Variations in the circadian rhythm are associated with the occurrence of a depression diagnosis and the severity of depressive symptoms. Moreover, in previous research there is evidence that markers of circadian rhythm (i.e., chronotype, melatonin levels etc.) can be used as a predictor of response in the treatment of depression. Implementing these markers in the clinic would be beneficial for providing more individualized treatment options for patients. In this systematic review an oversight will be given on the current status of evidence for the use of circadian markers for predicting the effect of treatment in depression. **Methods**: Multiple databases were searched for research studies or articles, randomized control trials and case report/series (EMBASE, PUBMED, PSYCHINFO). Other criteria that were used included that the effect of an antidepressant therapy had to be studied, the effect had to be measured by the level of depressive symptoms and/or occurrence of a clinical diagnosis of depression and a marker of circadian rhythm had to be assessed at baseline. **Results**: In total, 3980 articles were identified from the initial search, of which 42 relevant studies were included in the review. **Conclusion**: The discussion and conclusion of the systematic review has not been finished and will be presented at the symposium.

**Keywords**: circadian rhythm; chronotype; depression; systematic review

### 2.13. Seasonality of Human Sleep-I: Influence of Individual Melatonin Levels in Healthy Subjects

Dieter Kunz, Amely Wahnschaffe, Nina Kaempfe and Richard Mahlberg

Clinic Sleep- & Chronomedicine, St. Hedwig-Hopital, Berlin, Germany

**Abstract: Background**: The pineal hormone melatonin is the natural transducer of the environmental light–dark signal to the body. Although the responsiveness to photoperiod is well conserved in humans, only some 25 percent of the human population experiences seasonal changes in behavior. As a consequence, humans seem to have adapted—at least partly—to the seasonal changes in day length. The aim of the study was to demonstrate that the individual melatonin-deficit marker DOC (degree of pineal calcification) is related to variation of seasonal phenomena in humans. **Methods**: Out of 3011 patients in which cranial computer tomography (cCT) was performed for diagnostic reasons, in the end 97 consecutive ‘healthy’ subjects (43 female, 54 male; age 18–68 years, mean ± SD: 35.0 ± 13.1) were included. Exclusion criteria were, e.g., pathological finding in cCT, acute/chronic illness including alcohol/drug abuse, shift work, medication known to influence melatonin excretion. Degree of pineal calcification DOC was semi-quantitatively determined using the previously validated method. The seasonal pattern assessment questionnaire (SPAQ) was performed in a telephone interview. **Results**: Twenty-six subjects fulfilled criteria for seasonal affective disorder (SAD) or subsyndromal (S)-SAD. Seasonality was more pronounced in women as compared to men (SPAQ seasonality score 7.8 ± 4.0 vs. 4.9 ± 4.5; *p* = 0.001) and negatively and significantly associated with age (r = −0.178; *p* = 0.04). Subjective sleep length significantly varied between seasons (one-way repeated measures ANOVA: F = 45.75; *p* < 0.0001) with sleep during winter being 53 min (±70 min) longer than during summer. Controlling for age, total seasonality score was negatively and significantly associated with DOC (r94 = −0.214; *p* = 0.036). **Discussion**: Data confirm earlier studies with respect to distribution of seasonality with sex and age. The survival of seasonality in sleep length of people living in an urban environment underlines functionality of the circadian timing system in modern societies. Moreover, data confirm for the first time that diminished experience of seasonality in behavior is associated with a reduced individual capacity to produce melatonin. We have earlier shown the degree of pineal calcification (DOC) to be an individual melatonin deficit marker. Increased DOC scores are associated with disturbances of circadian functionality in patients with such diverse disorders as Alzheimer’s disease and insomnia. High DOC scores may represent an indicator for melatonin replacement therapy. The data presented here add the lack of seasonality to the group of circadian disturbances associated with DOC. Melatonin is the most robust human hormone, being suppressed only by light or the use of betablocker, but unaffected by, e.g., stress, sleep deprivation or a long scale of substances. Still, since low melatonin levels were not consistently associated to disturbances, a definition for hypopinealism does not exist. We suggest high DOC scores associated with disturbances of circadian functionality to constitute the state of hypopinealism. The mechanisms behind the calcification process of the pineal gland are not understood. Possibly, because among all living creatures studied today humans show the most pronounced calcified pineal gland, pineal calcification represents a human adaptation to modern life.

### 2.14. Seasonality of Human Sleep-II: PSG-Data in Neuropsychiatric Sleep Patients

Aileen Seidel, Jan de Zeeuw, Frederik Bes and Dieter Kunz

Clinic Sleep- & Chronomedicine, St. Hedwig-Hospital, Berlin, Germany

**Abstract: Introduction**: Earth rotation causes precise 24 h changes in the environment. Evolutionary adaptations have created circadian systems that anticipate these changes with light and darkness being the strongest zeitgebers. Except close to the equator, the length of the light–dark signal varies over the year, triggering seasonal phenomena such as breeding, migration and hibernation. In humans, photoperiod responsiveness is well conserved, but seems to be more complex. Seasonal changes in physiology differ according to age, sex, light source and many more variables, including some 70 percent of the population not reporting any seasonal changes in behavior. We have recently replicated this complexity in healthy subjects and in the same population reported human seasonality to be influenced by changes in intraindividual melatonin secretion levels (Seasonality of Human Sleep-I: this meeting). The aim of this study was to investigate seasonality of human sleep architecture. **Methods**: Three consecutive nights of polysomnography were performed in 292 patients in the Clinic for Sleep- & Chronomedicine, St. Hedwig-Hospital, Berlin in 2019. Diagnostic second nights were averaged per month and analyzed over the course of one year. Patients were advised to sleep ‘as usual’ including timing; alarm clocks were not allowed. Exclusion criteria were: administration of psychotropic agents known to influence sleep (n = 96), REM-sleep latency < 120 min (n = 5), technical failure (n = 3). Included were 188 patients: 46.6 ± 15.9 years (mean ± SD; range 17–81 years); 52% female. Sleep-related diagnoses were: insomnia (n = 108), depression (n = 59), sleep-related breathing disorders (n = 52), RLS (n = 20), PLMD (n = 36), RBD (n = 19) and others (n = 15). Diagnostic second nights were averaged per month and analyzed over the course of one year. For statistical analyses, a linear-mixed-model was performed. **Results**: Analyses showed significant differences in: 1. sleep-period time, with longer sleep during winter compared to summer (80 min; *p* = 0.036); 2. higher amount of REM sleep during winter compared to summer (30 min; *p* = 0.008); 3. shorter REM-sleep latency during winter compared to summer (22 min, *p* = 0.012); and 4. shorter NREM-3 in autumn (40 min; *p* = 0.044). **Conclusions**: To the best of our knowledge the data present the first evidence of seasonal variation in human sleep architecture in a heterogenous group of patients with disturbed sleep. The mechanisms behind this are still poorly understood. Nevertheless, these findings emphasize the extent to which humans are subject to seasonal changes even when living in an urban environment and may add to the ongoing discussion of daylight-saving time.

### 2.15. The Post-Illumination Pupil Response Correlates with Cognition in REM-Sleep Behavior Disorder

Oliver Steiner, Jan de Zeeuw, Sophia Stotz, Frederik Bes and Dieter Kunz

Sleep Research & Clinical Chronobiology, Institute of Physiology, Charité-Universitätsmedizin Berlin, Berlin, Germany

**Abstract: Background**: Isolated REM-sleep behavior disorder (iRBD) represents a prodromal phase of α-synucleinopathies such as Parkinson´s disease and Lewy body dementia. As a part of the brain, the retina may provide a window into the brain, including neurodegenerative processes. The pupil light response has been shown to be impaired in Parkinson´s disease. We analyzed the pupil light response in iRBD patients to determine whether chromatic pupillometry metrics could be used as biomarkers for cognitive deterioration in iRBD patients. **Methods:** In a cohort of 69 iRBD patients (14 female, age: 67 ± 9 years; mean ± SD), we compared chromatic pupilometry metrics with cognition. The pupil light response was measured after 10 min of dark adaptation, following a 1 s blue light pulse (56 cd/m^2^; peak wavelength at 463 nm). We determined the minimum pupil size (MPS) and the post-illumination pupil size at 6 s after the light pulse (PIPR) relative to the baseline pupil size. Cognition was assessed with the CERAD-Plus test battery. Each cognitive test was analyzed separately and two composite scores were calculated, one for global cognition and one for executive functioning. Ten patients were diagnosed with mild neurocognitive disorder (mNCD). Fifty-one patients also underwent dopamine-transporter single-photon emission computed tomography (DaT-SPECT). A z-score in the posterior putamen 2 SD below age-matched healthy reference was defined as low DaT density. **Results**: iRBD Patients with mNCD had significantly reduced MPS (*p* = 0.010) and PIPR (*p* = 0.001) compared to patients without mNCD. PIPR amplitude was significantly correlated with global cognition (r = 0.292, *p* = 0.015) and executive functioning (r = 0.417, *p* < 0.001). MPS did not show significant correlation with either cognition composite score (*p* > 0.050). In 26 patients with low DaT density, the significant correlations between PIPR and cognition were stronger (global cognition: r = 0.544, *p* = 0.004; executive functioning: r = 0.575, *p* = 0.002). **Conclusions**: We found significant associations between the PIPR and cognition in iRBD patients. This indicates an association between melanopsin function and cognition in iRBD since the PIPR is mainly controlled by melanopsin-expressing intrinsically photosensitive retinal ganglion cells, while MPS is mainly controlled by rods and cones. That this association was stronger in patients with low DaT density, which is known to represent an advanced state of iRBD, suggests that the PIPR can be used as a marker of cognitive deterioration in prodromal α-synucleinopathies. Correspondingly, we found the strongest correlation between PIPR and executive functioning, which is the cognitive domain that has been shown to be particularly impacted in Parkinson´s disease. Since chromatic pupillometry is non-invasive, inexpensive and easy to use, it may be a valuable new tool in clinical settings or in studies on disease progression of α-synucleinopathies.

### 2.16. Timescale Variation of Daytime Complaints Associated with Sleepiness and Fatigue among Patients Suffering from Obstructive Sleep Apnea and Narcolepsy

Vaida Verhoef ^1^, Karin Smolders ^1^, Geert Peeters ^2^, Sebastiaan Overeem ^1^, Yvonne de Kort ^1^

^1^  Industrial Engineering and Innovation Sciences, Eindhoven University of Technology, Eindhoven, The Netherlands
^2^  Kempenhaeghe Sleep Center, Heeze, The Netherlands

**Abstract: Background**: Patients diagnosed with sleep disorders like obstructive sleep apnea or narcolepsy often experience daytime consequences of their disturbed sleep. Excessive daytime sleepiness (EDS), chronic fatigue or tiredness are the most concerning, along with other cognitive and behavioral consequences. The interrelated nature of the symptoms and the overlap between the terms used to describe them render their diagnosis and treatment difficult. Several studies have shown that light exposure during the day can benefit people by improving levels of alertness, vitality and mood and by decreasing levels of experienced daytime sleepiness. However, these results do not form a consensus and the application of light exposure for therapeutical use in patients with daytime complaints such as EDS or fatigue is not yet widely established. Inconsistencies about the beneficial effects of light have been linked to various arguments, two of them being the timing of the exposure and the uncertainty of the state (alertness, sleepiness, tiredness, fatigue) it should and can target. The aim of this study was to capture the first-hand experiences of patients suffering from sleep apnea and narcolepsy, in order to investigate possible daytime complaints and their variations. **Methods**: Twenty patients (20–74 years old, 12 females and 8 males) of Dutch nationality, suffering from sleep apnea (n = 15) or narcolepsy (n = 5), were invited to semi-structured interviews. The questions were focused on daytime complaints linked with their diagnosis, their variations throughout and across days, and possible solutions and strategies implemented by patients. The transcribed answers were analyzed using an eclectic coding method and a thematic analysis. **Results**: Analyses showed the prominence and prevalence of sleepiness, fatigue or tiredness among the daytime complaints of patients, and elucidated their everyday phenomenology. All three interrelated concepts were defined and described differently by patients, with the use of metaphors or a clear distinction between physical and mental symptoms. Interestingly, the experiences related to sleepiness, fatigue or tiredness did not show a consensual temporal pattern across all participants. Indeed, the thematic analysis uncovered a distinction between constant subjacent states and sudden occurrences, as well as a distinction between linear time variation and curvilinear patterns as a function of time of day. While some expressed a worst state of sleepiness, fatigue or tiredness in the morning with an improvement throughout the day, others stated the opposite, or mentioned ups and downs in their complaints. **Conclusions**: The preliminary outcomes from the present qualitative study inform us of the individual nature of daytime complaints related to diagnosis of sleep disorders, and their temporal pattern across the day. The diversity of the complaints as well as of the time variations indicates a need for individually timed (light) interventions, for repeated assessments across multiple aspects of health (not just sleepiness), and for more individual care trajectories. 

**Keywords**: daytime sleepiness; fatigue; sleep apnea; narcolepsy; daytime complaints

**Funding:** This research is part of the European Training Network LIGHTCAP (project number 860613) supported by the Marie Skłodowska-Curie actions framework H2020-MSCA-ITN-2019.

### 2.17. Ultra-High-Field 7 Tesla fMRI Potential in Revealing Hypothalamus Responses to Blue-Enriched Light Exposure

Roya Sharifpour ^1^, Islay Campbell ^1^, Ilenia Paparella ^1^, Elise Beckers ^1^, Fermin Balda ^1^, Nasrin Mortazavi ^1^, Alexandre Berger ^2^, Puneet Talwar ^1^, Ekaterina Koshmanova ^2^, Laurent Lamalle ^1^, Christophe Philips ^1^, Siya Sherif ^1^, Gilles Vandewalle ^1^

^1^  Sleep and Chronobiology Laboratory, GIGA-Institute, CRC-In Vivo Imaging Unit, University of Liège, Liège, Belgium
^2^  Institute of Neuroscience (IoNS), Université Catholique de Louvain (UCLouvain), Louvain, Belgium

**Abstract: Introduction**: In addition to vision, light serves many non-visual effects including the stimulation of alertness and cognition. These non-visual effects are mainly mediated through photoreceptors known as intrinsically photosensitive Retinal Ganglion Cells (ipRGCs) and expressing the melanopsin photopigment. While the hypothalamus is known as a primary target of ipRGCs, the precise workings of its numerous nuclei that receive direct and indirect ipRGCs inputs are not established. Here, we investigated the potential of high-resolution ultra-high-field (UHF) 7 Tesla (7T) MRI to provide insight into the roles of the nuclei of the hypothalamus in mediating the non-visual impact of light on alertness and cognition. **Method**: We recorded 19 healthy young participants (22–30 years old; 10 women; ongoing data acquisition) with 7T functional MRI; they were asked to perform an auditory working memory task (N-back) and alternately were maintained in darkness or were exposed to 30 s light blocks which were composed of either blue-enriched light (100, 200 and 400 microWatt/cm^2^, for which melanopic lux were 63, 155, 310, respectively) and monochromatic orange light (1013 photons/cm/s; mostly undetected by ipRGCs, for which melanopic lux was 0.2). MRI dat-processing included data-driven/machine-learning segmentation of the hypothalamus into several sub compartments, gathering a few nuclei only. Results: Preliminary results suggest a blue-enriched-light impact in the so-called inferior tubular part of the hypothalamus (uncorrected *p* < 0.005), which includes the lateral hypothalamus (LH), key to sleep–wake regulation, and the tuberomammillary nucleus (TMN), one of the alertness centers in the brain which controls arousal. Moreover, our preliminary results show a blue-enriched-light modulatory impact bilateral in the superior tubular part of the hypothalamus (uncorrected *p* < 0.008), which includes the lateral hypothalamus (LH), and dorsomedial nucleus (DMN), essential relay of the suprachiasmatic nucleus, which is the site of the master circadian clock. Conclusions: These results show that UHF 7T MRI is promising in unraveling the subcortical wiring of the non-visual impact of light, including at the level of the hypothalamus. Future analyses on a larger sample size will test whether other hypothalamus compartments are affected by light and will assess cross-talk between nuclei. Support: FNRS, ULiège, EU FEDER program, Fondation Léon Frédéric, LIGHTCAP EU-ETN-MSCA, ULiège Innovation Chair. 

### 2.18. Influence of Chronotype of People on Antihypertensive Therapy

Yklym Bolmammedov and Taganmyrat Hojageldiyev

Turkmen State Medical University named after Myrat Garryev, Ashgabat, Turkmenistan

**Abstract: Introduction**: As many biological processes, blood pressure is also modulated in a circadian rhythm; normally it dips by 10% to 20% at night (O’Brien E. et al., 1988). There are several factors that contribute to the non-dippers and abnormal nighttime blood pressure. Here, we suggest that chronotype of patients may play the main role in response to anti-hypertensive drugs by the differing prevalence of non-dippers. **Materials and methods**: Forty-two patients with controlled hypertension who were taking same anti-hypertensive therapy according to unique treatment protocol were evaluated with 24 h blood-pressure monitoring. Non-dippers are defined as a systolic or diastolic blood-pressure nocturnal drop of less than 10%. The Horne–Ostberg questionnaire was used to assess the chronotype of patients. The analysis was done using Graph Pad Prism 7. **Results**: According to questionnaire responses, 33 (78.6%) were ‘morning-type’, 9 (21.4%) were ‘neither-type’ and there were no evening-type patients. According to blood pressure rhythm, in ‘morning-type’ patients, 45.5% showed a dipper blood-pressure pattern and 54.5% showed a non-dipper blood-pressure pattern. In neither-type patients, 33.3% showed a dipper blood-pressure pattern and 66.7% showed a non-dipper blood-pressure pattern. **Conclusion**: In contrast to the same anti-hypertensive treatment algorithm for both chronotypes, the efficiency of treatment was different. The number of patients with non-dipper blood pressure pattern was higher in ‘evening-type’ patients. In the future, a more detailed study will provide evidence for the suggestion that the chronotype of people may serve as a proxy for drug response.

### 2.19. Comparison of Academic Achievements of Students with Different School Shifts According to Their Chronotype

Yklym Bolmammedov and Taganmyrat Hojageldiyev

Turkmen State Medical University named after Myrat Garryev, Ashgabat, Turkmenistan

**Abstract:** There are many factors that influence students’ academic performance such as socioeconomic status, parents and quality of teachers. Biological factors that affect students’ grades are sleep and chronotype. The personalized biological circadian rhythm known as a ‘chronotype’ is expressed as an optimal time to fall asleep and wake up. Previously conducted studies revealed the effects of desynchronization of internal and social clocks on health and physical and mental performance. Early chronotype has been linked to physical and mental health, self-esteem, school functioning and intimate relationships, while late chronotype has been shown to be associated with mental illness, infections, smoking and poor sleep quality (Zerbini, G., & Merrow, M. 2017). There are still some gaps in studies seeking more productive school, work or training hours, despite numerous studies conducted for evaluating the impact of a students’ chronotype on academic performance. Here, we studied the academic performance of school students on different shifts according to their chronotype. **Materials and methods**: The study included 350 students of 10–11th grade (age 17–18) in secondary schools in Ashgabat for each of three different shifts. School starting hours for morning shift 08:30 a.m., for evening shift 13:30 PM and for state boarding school with an extended shift with 90 min planned afternoon napping for 08:30 a.m. All participants were tested using the Morningness–Eveningness Questionnaire to assess chronotype such as morning (MC), evening (EC) and neither chronotype (NC). School success of students was determined by the average grade in the last semester. Statistical analysis were performed using GraphPad Prism 7.0 and with a *p*-value < 0.05 considered significant. **Results**: According to test responses, percentile distribution of chronotypes of students and their average grades (in brackets) were as follows: morning shift MC-48.3% (4.15), EC-2.9% (3.84), NC-48.8% (3.97); evening shift MC-40.3% (3.90), EC-4.6% (3.97), NC-55.1% (3.88); napping shift MC-46.3% (4.03), EC-3.4% (3.96), NC-50.3% (4.11). Distribution of chronotypes of the students in each shift is not significant and no gender bias exists. Significant differences in grades of students was revealed between MC and EC in morning and evening shifts. Students of napping shift had overall better academic performance for all chronotypes, where NC had notably higher grades than NC students of other shifts. **Conclusion**: Our findings that early chronotypes perform better in morning shifts and late chronotypes do their best in evening shifts are in line with previous studies. Afternoon napping improves students’ academic performance by not affecting the temporal phenotype of students. Further extended researches on napping, sleep quality and circadian preferences of students will help to create more productive class schedules that will improve school performance.

### 2.20. Reduced Fall Rate in Elderly Care Home Residents Following Installation of Solid-State Dynamic Lighting

Leilah Grant ^1^, Melissa St. Hilaire ^1^, Jenna Heller ^2^, Rodeny Heller ^2^, Steven Lockley ^1^ and Shadab Rahman ^1^

^1^  Brigham and Women’s Hospital, Harvard Medical School, Boston, MA, USA
^2^  Midwest Lighting Institute Inc., Cottage Grove, WI, USA

**Abstract: Background**: Falls are the leading cause of injury-related death in adults aged 65 years and over in the United States. Improved lighting positively influences several important factors that contribute to fall risk, including cognitive impairment, sleep disturbance and visual acuity; lighting interventions to directly reduce falls have yet to be tested, however. We aimed to test whether the implementation of a solid-state dynamic lighting system is associated with a reduction in fall rate in long-term care home residents as compared to typical static lighting. **Methods**: The number of falls for 758 residents (mean age (±SD) 81.0 ± 11.7 years; 57% female; 31% with dementia) were retrospectively assessed from medical records over a 2-year interval at two pairs of care homes. One ‘experimental’ site from each pair had solid-state lighting installed throughout the facility, which changed in intensity and spectrum to increase exposure to higher intensity short-wavelength light during the day (6 am–6 pm) and decrease it overnight (6 pm–6 am). The ‘control’ site retained standard static lighting with no change in intensity or spectrum. The primary outcome was the rate of falls/1000 resident days. **Results**: In the year prior to the lighting upgrade, the fall rate was similar between the control and experimental sites (6.94 vs. 6.62 falls/1000 resident days, respectively; RR 1.05; 95% CI 0.70, 1.58; *p* = 0.82). Following the upgrade, falls were reduced by 43% at experimental sites compared to control sites (4.82 vs. 8.44 falls/1000 resident days, respectively; RR 0.57; 95% CI 0.39, 0.84; *p* = 0.004) and remained significant after adjusting for age, sex and dementia. **Conclusions**: Implementation of a solid-state dynamic lighting system may represent an effective, passive, non-invasive and relatively inexpensive intervention to reduce fall risk in long-term care settings. 

**Funding:** Civil Money Penalty Funds awarded to the Midwest Lighting Institute, Ltd. by the State of Wisconsin Department of Health Services, Division of Quality Assurance.

### 2.21. Daytime Alertness, Mood and Cognition Improved by Supplementing Sub-Optimal Ambient Lighting with a High-Melanopic Illuminance Task Lamp

Leilah Grant ^1^, Phoebe Crosthwaite ^2^, Matthew Mayer ^2^, Robert Stickgold ^1^, Steven Lockley ^1^ and Shadab Rahman ^1,3^

^1^  Harvard Medical School, Brigham and Women’s Hospital, Boston, MA, USA
^2^  Brigham and Women’s Hospital, Boston, MA, USA
^3^  Harvard Medical School, Beth Israel Deaconess Medical Center, Boston, MA, USA

**Abstract: Background**: Although ambient indoor lighting with high melanopic illuminance has been shown to improve alertness and neurobehavioral performance, it is unknown whether supplementing sub-optimal ambient lighting with a high melanopic task lamp has similar benefits. In the current study, we therefore examined the impact of a single high melanopic illuminance supplemental task lamp on alertness, mood and cognition during a simulated workday. **Methods**: Sixteen healthy young adults (mean ± SD age = 24.2 ± 2.9 years, 8F) completed a 3-day inpatient crossover study. All participants were mildly sleep restricted (7 h time-in-bed nightly) during the two inpatient nights and for 7 days prior to the study. On days 2 and 3 of the inpatient study, participants underwent an 8 h simulated workday during which they were randomly assigned to one of two light conditions: (1) ambient fluorescent indoor lighting (~30 melanopic lux, measured in the vertical plane at a fixed location at approximate eye level) and (2) ambient indoor lighting supplemented with high-melanopic content lighting from an LED task lamp (~250 melanopic lux). Alertness, mood and cognitive performance were assessed throughout the light exposure and compared between conditions using linear mixed models. **Results**: Participants exposed to the high-melanopic task lamp reported less sleepiness on the Karolinska Sleepiness Scale (*p* = 0.01), in addition to feeling more alert, happy, healthy, energetic, mentally sharp and motivated (all *p* ≤ 0.02), as measured by Visual Analog Scales. Furthermore, the percentage of correct responses on a 2 min addition task was higher (*p* < 0.001), the Psychomotor Vigilance Task (PVT) reaction time was faster (*p* = 0.009) and lapses (>500 ms) were lower (*p* = 0.02) when exposed to the supplemented lighting. **Conclusions**: Subjective alertness, mood and objectively assessed neurobehavioral performance were improved with high melanopic illuminance task lighting. Incorporation of supplemental task lighting into existing sub-optimal lighting environments may benefit performance without the need for resource-intensive remodeling.

**Funding:** This work was supported by an investigator-initiated grant from Biological Innovation and Optimization Systems, LLC.

### 2.22. Office Lighting and Cognitive Functions: Can It Be Too Bright

Marta Benedetti ^1^, Lenka Maierova ^2^, Christian Cajochen ^3^, Jean-Louis Scartezzini ^1^, Mirjam Münch ^4^

^1^  Solar Energy and Building Physics Laboratory (LESO-PB), Ecole Polytechnique Fédérale de Lausanne (EPFL), Vaud, Switzerland
^2^  University Centre for Energy Efficient Buildings (UCEEB), Czech Technical University in Prague, Prague, Czech Republic
^3^  Centre for Chronobioloy, University Psychiatric Clinics Basel, Basel, Switzerland
^4^  Centre for Chronobiology, University Psychiatric Hospitals, Basel, Switzerland; Transfaculty Research Platform Molecular & Cognitive Neurosciences, University of Basel, Basel, Switzerland

**Abstract**: **Background**: Innovative daytime lighting conditions can impact wellbeing and productivity and enhance cognitive functions in office workers. This study aimed to test whether optimized office lighting over several days increases vigilant attention, reaction time and working memory. **Methods**: Thirty-four young participants spent 5 consecutive days (for 8 h) in an office room equipped with an automated controller for blinds and electric lighting and a larger window surface (= test room). Separated by one week, they also spent 5 consecutive days in a control office room without a controller (= reference room) in a balanced-crossover design. Every 2.5 h, participants had to complete an auditory cognitive test battery containing the Psychomotor-Vigilance Test (PVT) and the 0-2-3-back task with spoken letters, followed by subjective assessments of mood, alertness, temperature and glare. Light exposures were assessed with stationary devices for photopic illuminance and irradiance. **Results**: Photic illuminance in a vertical plane at eye level was, on average, 320 lux higher in the test than the reference room reported recently, which corresponded to a difference in melanopic EDI of ≈200 lux (Benedetti et al. 2022). Unexpectedly, reaction times assessed during the PVT (median, 10% fastest and 10% slowest) were significantly slower in the test than in the reference room (the difference was on average 7.5 ms for the 10% slowest; main effect of room condition; generalized mixed models; *p* < 0.05). Similarly, accuracy in the 2-back test was worse in the test room compared to the reference room (*p* < 0.05). There was no statistically significant difference in the more difficult 3-back test or in the simple 0-back test between the test and reference room. Subjective alertness did not differ between both rooms’ lighting conditions, nor did subjective effort in the tasks, or perception of temperature or subjective glare. However, mood and objective glare assessments (determined by the Daylight Glare Probability index) were slightly worse in the test than the reference room (*p* < 0.05), even though both variables remained clearly within the better mood and imperceptible glare half of the evaluation spectrum. **Conclusion**: The office lighting with an automated control system did not lead to better cognitive performance when compared to the reference room, most likely because in the reference room (with approximately 730 melanopic EDI (lux)), the lighting was already far above the minimum recommended daytime lighting levels (Brown et al., 2022). The worsening of cognitive performance in the test room points to a tentative inverted u-shape for light effects on cognitive performance, where too low and too bright lighting might impair cognitive performance. Combinations with other factors such as room temperature (which was on average 0.8 °C lower in the test than the reference room) and season may also play a role, and the personalized ‘optimum’ lighting for cognitive functions still needs to be determined. Most studies focusing on lights’ impact on biological functions and cognitive performance compared rather low with brighter lighting. Hence, there is a need to explore the upper lighting thresholds for these functions to define the optimal dose of light in the office.

### 2.23. Light Exposure Behavior Assessment (Leba): A Novel Self-Reported Instrument to Measure Light Exposure-Related Behavior

Mushfiqul Anwar Siraji ^1^, Rafael Robert Lazar ^2^, Juliëtte van Duijnhoven ^3^, Luc Schlangen ^4^, Shamsul Haque ^5^, Vineetha Kalavally ^6^, Céline Vetter ^7^, Gena Glickman ^8^, Karin Smolders ^9^ and Manuel Spitschan ^10^

^1^  Jeffrey Cheah School of Medicine and Health Sciences, Monash University, Melbourne, Australia
^2^  Centre for Chronobiology, Psychiatric Hospital, University of Basel, Basel, Switzerland
^3^  Built Environment, Building Lighting, Eindhoven University of Technology, Eindhoven, The Netherlands
^4^  Industrial Engineering and Innovation Sciences, Eindhoven University of Technology, Eindhoven, The Netherlands
^5^  Jeffrey Cheah School of Medicine and Health Sciences, Monash University, Melbourne, Australia
^6^  lectrical and Computer Systems Engineering, Monash University, Melbourne, Australia
^7^  University of Colorado Boulder, Boulder, CO, USA
^8^  Uniformed Services University of the Health Sciences, Bethesda, MD, USA
^9^  Industrial Engineering and Innovation Sciences, Eindhoven University of Technology, Eindhoven, The Netherlands
^10^  Max Planck Institute for Biological Cybernetics, Tübingen, Germany

**Abstract: Background**: Light exposure is essential for our health and well-being, driving various non-visual processes, including circadian photoentrainment, melatonin suppression and the modulation of alertness. An unexplored dimension of light exposure is that it is partially controlled by our behavior. Here, we present a novel instrument to capture light exposure-related behavior: the Light Exposure Behaviour Assessment (LEBA). **Methods**: An expert panel prepared an initial item pool (n = 48 items). Responses, consisting of rating the frequency of engaging in a given behavior on a five-point Likert scale, were collected using a fully anonymous, geographically unconstrained online survey (n = 690 complete responses across 74 countries and 28 time zones). Five different attention-check items were included in the survey to ensure high data quality. We applied a psychometric analysis strategy based on Classical Test Theory and Item Response Theory (IRT) to develop and validate the LEBA instrument. **Results**: In the exploratory factor analysis (EFA) on an initial subset of our sample (n = 428), a five-factor latent structure with 25 items was obtained (F1: wearing blue light filters; F2: spending time outdoors; F3: using phone and smart watch in bed; F4: using light before bedtime; F5: using light in the morning and during daytime). A subsequent confirmatory factor analysis (CFA) was performed on another independent subset of participants (n = 262) to assess structural validity. The final CFA analysis yielded a five-factor latent structure with 23 items (CFI = 0.95, TLI = 0.95 and RMSEA = 0.06). The five factors’ internal consistency reliability coefficient ordinal alpha ranged between 0.52 and 0.96, indicating sufficient to good internal consistency. The full scale also showed adequate internal consistency (McDonald’s Omega = 0.73). Our model also exhibited the highest level of invariance–residual invariance between native and non-native English speakers (CFI = 0.95, TLI = 0.95, RMSEA = 0.05). Finally, we used IRT on the complete sample (n = 690) to develop a short form of LEBA (18 items), excluding five items carrying low information to distinguish individuals in terms of their attitude towards light hygiene. **Conclusion**: The psychometric properties of both forms of LEBA indicate the potential usability for measuring individuals’ general light exposure-related behavior at a large scale. The LEBA instrument may offer a scalable solution to characterize persons based on their engagement in these behaviors, which may help to develop interventions for optimizing personal light exposure. Adaptations of LEBA into Bangla and German are currently in preparation. The LEBA instrument will be available under the open-access CC-BY-NC-ND license (https://leba-instrument.org/ accessed on 28 February 2022).

**Keywords**: light exposure; light-related behavior; non-visual effects of light; psychometrics

**Funding:** This research was supported by funding from the Welcome Trust (204686/Z/16/Z), the European Training Network LIGHTCAP (project number 860613) under the Marie Skłodowska-Curie actions framework H2020-MSCA-ITN-2019, the BioClock project (number 1292.19.077) of the research program Dutch Research Agenda: Onderzoek op Routes door Consortia (NWA-ORC) which is (partly) financed by the Dutch Research Council (NWO), and the European Union’s Horizon 2020 programme and the nationals contributing in the context of the ECSEL Joint Undertaking (JU) under grant agreement No 101007319.

### 2.24. Measurements of Retinal Spectral Exposure in Occupied Settings

David Sliney

Johns Hopkins University School of Public Health, Baltimore, MD, USA

**Abstract:** The directly measured spectral irradiance from lamps and luminaires are the traditional starting points for assessment of melanopic exposure in occupied settings. However, the spectral distribution is altered by the spectral reflectance of viewed areas. To measure the actual retinal exposure and its spectral distribution, a Gigahertz–Optik field-portable illuminance meter and spectroradiometer was fitted with a conical baffle in order to measure a localized brightness of the visual field being viewed. Most reflecting surfaces viewed and measured demonstrated a reduced spectral brightness at the spectrally important shorter visible wavelengths of the melanopic response as defined in the international standard, CIE S 026:2018 (CIE System for Metrology of Optical Radiation for ipRGC Influenced Responses to Light). More realistic measures of retinal exposures can be achieved by simple adaptations of common light-measurement instruments that can be used both in both the indoor lit environment and the outdoors environment. 

### 2.25. Chronotherapy in Adult ADHD: Results from the PhASE Study

Emma van Andel ^1^, Denise Bijlenga ^2^, Suzan Vogel ^1^, Aartjan Beekman ^3^ and Sandra Kooij ^4^

^1^  PsyQ Expertise Center Adult ADHD, Den Haag, The Netherlands
^2^  PsyQ Expertise Center Adult ADHD, Den Haag, The Netherlands; Sleep-Wake Center SEIN, Heemstede, The Netherlands
^3^  Amsterdam UMC, VU Amsterdam, Psychiatry, Amsterdam Public Health Research Institute, Amsterdam, The Netherlands; GGZ inGeest Specialized Mental Health Care, Amsterdam, The Netherlands
^4^  PsyQ Expertise Center Adult ADHD, Den Haag, The Netherlands; Amsterdam UMC, VU Amsterdam, Psychiatry, Amsterdam Public Health Research Institute, Amsterdam, The Netherlands

**Abstract: Objective**: The majority of adults with Attention-Deficit/Hyperactivity Disorder (ADHD) have a delayed circadian rhythm that is a characteristic of Delayed Sleep Phase Syndrome (DSPS). Treatment of DSPS may improve the circadian rhythm, sleep and ADHD symptoms. **Methods**: In this three-armed randomized clinical trial, 51 adults (18–55 year) with ADHD and DSPS received sleep education and 3 weeks of (1) 0.5 mg/d placebo, (2) 0.5 mg/d melatonin or (3) 0.5 mg/d melatonin plus 30 min of 10,000 lux bright light therapy (BLT) between 07:00 and 08:00 h. Placebo and melatonin conditions were double-blind, and treatment took place in the participants’ naturalistic home settings. Dim-light melatonin onset (DLMO) was measured in saliva as marker of internal circadian rhythm. Melatonin or placebo administration followed individual schedules, starting 3 h before the individual DLMO and weekly advancing by one hour. DLMO, ADHD Rating Scale score and sleep were assessed at baseline, directly after 3-week treatment, and two weeks after the end of treatment. Sleep was measured objectively and subjectively by actigraphy and questionnaires, respectively. **Results**: At baseline, 77% had a DLMO after 21:00 h with an average DLMO at 23:43 h ± 1 h 46. Directly after treatment, melatonin had advanced DLMO by 1h28 (*p* = 0.001), and melatonin plus BLT by 1h58 (*p* < 0.001). Placebo did not affect DLMO. ADHD symptoms decreased by 14% (*p* = 0.062) directly after melatonin treatment. Two weeks after end of treatment, ADHD symptoms and DLMO had returned to baseline levels. Placebo did not impact ADHD symptoms. Neither did melatonin plus BLT, which could be due to BLT timing being fixed rather than individualized. The interventions did not advance sleep times, improve sleep in general or strengthen wake-activity rhythms. **Conclusion**: Low doses of melatonin advanced the circadian rhythm and reduced self-reported ADHD symptoms. Given the large number of adult ADHD patients with concurrent DSPS, treating delayed sleep with melatonin is an important component of effective ADHD treatment. Sleep, however, did not advance or improve, despite people being biologically prepared for sleep. More extensive behavioral coaching is necessary to change long-standing sleep behaviors, and BLT timing should be individualized. This way, sleep onset time and consequently wake time will advance along with DLMO, leading to longer sleep duration and better sleep in general, which may in turn further alleviate ADHD symptoms. 

### 2.26. Effects of Acute Light Exposure at Night on Behavior and Neuronal Activity in the Lateral Hypothalamus in the Diurnal Rodent Rhabdomys Pumilio

Asshen Dedigama Acharige, Joshua Mouland, Timothy Brown, Robert Lucas and Beatriz Bano-Otalora

University of Manchester, Manchester, UK

**Abstract:** The natural relationship between ambient light intensity and activity/arousal across the 24-hour day is inverted in animals occupying nocturnal vs diurnal temporal niches. This places a limit on the utility of standard laboratory rodents (which are all nocturnal) as models for understanding the link between light and arousal in humans. We therefore set out to explore the relationship between light and activity/arousal in the African four-striped mouse (Rhabdomys pumilio), a murid rodent that maintains high levels of activity in daylight in the field and remains robustly diurnal under laboratory lighting regimes. We applied 30 min light pulses at night (ZT16) to R. pumilio under stable entrainment to a 12:12 LD cycle and measured general activity and c-fos expression in orexinergic neurons of the hypothalamus. Comparison with baseline nights indicated that the 30 min light pulse could drive an increase in general activity. We did not detect a measurable increase in c-fos expression in orexinergic neurons following the light pulse, but there was a suggestion that c-fos was up-regulated in neighbouring non-orexinergic neurons in the lateral hypothalamus. We conclude that a nocturnal light pulse can produce increased activity in R. pumilio. Future work will be required to define the relationship between this behavioral response and the activity of neurons in the lateral hypothalamus.

### 2.27. Solid State Lighting Countermeasures to Improve Circadian Adaptation, Sleep and Performance during a High Fidelity Analog Study for the International Space Station

John Hanifin ^1^; Melissa St. Hilaire ^2^, John Kemp ^1^, Benjamin Warfield ^1^, Fadee Disoke ^1^, Talia Glodjo ^1^, Samar Jasser ^1^, Melissa Ayers ^1^, Leanna Panepinto ^1^, Sreeramya Kanumilli ^1^, Nicolas Nelson ^1^, Donna Hasher ^1^, Shalini Vadalia ^1^, John Balaicuis ^1^, Brenda Byrne ^1^, Carissa Pineda ^1^, Edward Gerner ^1^, Shadab Rahman ^2^, Steven Lockley ^2^ and George Brainard ^1^

^1^  Thomas Jefferson University, Philadelphia, PA, USA
^2^  Harvard Medical School, Brigham and Women’s Hospital, Boston, MA, USA

**Abstract: Background**: On space shuttle and International Space Station (ISS) missions, crewmembers obtained about one hour less sleep on nights with circadian misalignment [1,2]. Light can be a powerful countermeasure for both circadian misalignment and sleepiness. Originally, the ISS was equipped with fluorescent General Luminaire Assemblies (GLAs) for illuminating astronauts’ working and living environments. The majority of GLAs have been replaced with Solid State Lighting Assemblies (SSLAs) that emit three predefined color temperature settings [3]. The overall aim of the following work is to test light emitted by SSLAs for efficacy in supporting astronaut operational tasks, as well as regulating circadian, neuroendocrine, neurobehavioral and sleep physiology. **Methods**: A Dynamic Lighting Schedule has been developed based on the spectral and intensity sensitivity of the human circadian photoreceptor system. For this study, the SSLAs had three light settings, each with a unique intensity and spectrum to optimize their efficacy: (1) General Illumination 4500 K SSLA white light, 424 melanopic EDI; (2) Alertness/Phase Shift 6500 K SSLA (blue-enriched) white light, 1040 melanopic EDI; and (3) Pre-Sleep 2700 K SSLA (blue-depleted) white light, 10 melanopic EDI. Proper uses of the SSLAs are intended to: (1) facilitate circadian adaptation; (2) enhance sleep; and (3) improve alertness and performance. Part of the first specific aim of this project is to conduct an analog study using healthy astronaut-aged volunteers in a 5-day, randomized, controlled inpatient study to test the efficacy of a lighting protocol for daily operations that utilizes SSLAs. Those studies were conducted in the high-fidelity ISS analog crew quarters laboratory at Thomas Jefferson University. Results: Extensive data were collected on sleep, subjective alertness, neurocognition, plasma melatonin, urinary 6-sulfatoxymelatonin, visual performance and color vision. For example, dynamic lighting evoked significantly earlier evening onset of plasma melatonin. A mean of the three melatonin values taken prior to participants’ sleep opportunity in the evening of Day 4 showed that exposure to a dynamic lighting schedule produced higher plasma melatonin levels compared to exposure to a static lighting schedule (F = 64.32, *p* < 0.001). In addition, there were statistically significant differences (F = 9.48, *p* < 0.001) in color vision discrimination relative to the different light settings of the SSLAs. Conclusions: Risk factors for the health and safety of astronauts include disturbed circadian rhythms and altered sleep–wake patterns. Study results determine if SSLA lighting can be used to support astronaut vision as well as serve as an in-flight countermeasure for circadian misalignment, sleep disruption and performance deficits on the ISS. Appropriately designed lighting systems will serve to mitigate such risks in future exploration missions as well as in Earth-based applications. Key words: light, sleep, circadian, melatonin, spaceflight References: [1] Barger et al. (2014) Lancet Neurol 13, 904–912, [2] Flynn-Evans et al. (2016) NPG Micrograv 2, 1–6, [3] NASA Specification (2013) S684–13489. Support: Primary funding: NASA #NNX15AC14G. Additional support: NASA #NNX09AM68G, NSBRI through NASA NCC 9-58, NSF #EEC-0812056 and the Philadelphia Section of the IESNA.

### 2.28. Optimization, Working Mechanisms and Response Predictors of Bright-Light Therapy for Depression—A Randomized Multicenter Clinical Trial

Emma Visser ^1^, Oana Georgiana Rus-Oswald ^2^, Claudia Simons ^3^, Priscilla Oomen ^4^, Marijke Gordijn ^5,6^, Willem van der Does ^7^, Machteld Marcelis ^8^, Yvonne de Kort ^1^, Niki Antypa ^7^ and Luc Schlangen ^1^

^1^  Industrial Engineering and Innovation Sciences, Eindhoven University of Technology, Eindhoven, The Netherlands
^2^  Department of Clinical Psychology, Leiden University, Leiden, The Netherlands
^3^  GGzE Mental health institute of Eindhoven and the Kempen, Eindhoven, The Netherlands & Department of Psychiatry and Neuropsychology, School for Mental Health and Neuroscience, Maastricht University, Maastricht, The Netherlands
^4^  GGzE Mental health institute of Eindhoven and the Kempen, Eindhoven & Department of Biomedical Sciences of Cells and Systems and Department of Psychiatry, University of Groningen, University Medical Center Groningen, Groningen, The Netherlands
^5^  Chrono@Work B.V., Groningen, The Netherlands
^6^  Chronobiology Unit, Groningen Institute for Evolutionary Life Sciences, University of Groningen, Groningen, The Netherlands
^7^  Department of Clinical Psychology, Leiden University, The Netherlands
^8^  GGzE Mental health institute of Eindhoven and the Kempen, Eindhoven, The Netherlands & Department of Psychiatry and Neuropsychology, School for Mental Health and Neuroscience, Maastricht University, Maastricht, The Netherlands
^9^  Department of Clinical Psychology, Leiden University, The Netherlands

**Abstract: Background**: Bright-light therapy (BLT) is one of the first-line treatments for seasonal affective disorder, and is also used as an antidepressant treatment in other affective disorders. Despite evidence of its antidepressant efficacy, BLT is underused in clinical settings internationally. This might be due to a lack of knowledge of the underlying biological mechanism for the efficacy of BLT and the absence of clearly identified response predictors. Although BLT can restore observed circadian disturbances in patients with a depressive disorder by shifting the circadian phase, it is still unknown to what extent realignment of disturbed biological rhythms is a causal factor in the reduction of depressive symptoms. Other factors that are associated with the treatment, such as an altered social rhythm and increased social interaction, might be important contributors to the efficacy of the treatment. Additionally, it is still unknown whether BLT first increases sleep quality before reducing depressive symptoms or vice versa. **Aims**: We aim to conduct a randomized clinical trial to address three objectives: (1) optimize BLT with chronotherapeutic strategies and social rhythm interventions; (2) obtain more insight into the underlying mechanisms of BLT; and (3) identify potential response predictors of BLT. **Methods**: 240 patients (age: 18+) diagnosed with a depressive or bipolar disorder will be included and divided over three treatment arms. In all arms, patients will receive 10,000 lux BLT for 30 min per day for 5 consecutive days with a minimum of one week and a maximum of three weeks (depending on the remittance of depressive symptoms). In the first arm of the trial, patients will receive BLT in their home environment. In the second arm, BLT will be administered in a specialized clinic, under the supervision of clinical staff, promoting lifestyle changes and social interaction. In the third arm, treatment will be identical to the second arm but now complemented with the introduction of blue-light blocking glasses to be used in the evening and BLT treatment timing strategies based on sleep–wake patterns. Before and after treatment, dim light melatonin onset (DLMO) will be assessed to evaluate circadian phase-shifting effects of the treatment and to explore whether these correlate with a decrease in depressive symptoms. Ecological momentary assessment will be used to gain insight into the sequence of changes in energy levels, sleep and mood across treatment. Potential predictors of treatment response will be assessed before, during and after treatment and include baseline clinical characteristics, subjective and objective measures of sleep, circadian parameters (DLMO and melatonin suppression), and light-related behaviors.

**Keywords:** bright-light therapy; major depressive disorder; bipolar disorder; circadian rhythm; sleep; chronotype; interpersonal social rhythm therapy; blue-light blocking glasses; response predictors; ecological momentary assessment

**Funding:** This study is part of the project BioClock (with project number 1292.19.077) of the research program Dutch Research Agenda: Onderzoek op Routes door Consortia (NWA-ORC) which is (partly) financed by the Dutch Research Council (NWO).

### 2.29. Light, Activity and Sleep in My Daily Life: Design of an Online Intervention Targeting Changes to Routines and the Home

Kiran Maini Gerhardsson, Susanne Iwarsson and Steven Schmidt

Lund University, Lund, Sweden

**Abstract: Background**: Older adults spend more time at home after retirement, and the home becomes a central place for activity. While research indicates that indoor lighting, exposure to daylight, physical activity and sleep interact to influence functioning, mood and daily rhythm, strategies are needed to promote behavioral changes to optimize these factors in daily life. The objective is to design an intervention delivered as a web-based course to encourage behavior change related to outdoor physical activity, sleep patterns and changes to the home environment. The behavior changes are intended to promote mental wellbeing and improve lighting and darkness conditions. The intervention strategy departs from the Information–Motivation–Behavioral Skills Model. Intervention components build on goal implementation theory. The Technology Acceptance Model is used as a framework to evaluate usability aspects of the course content and the learning management system. **Method**: Using a mixed-methods approach, qualitative and quantitative data were collected through video observations, semi-structured interviews and a 10-item Likert scale questionnaire (The System Usability Scale). Scores were averaged for each participant and converted into a usability score out of 100 (a score of 68 or above is considered above average). In a first round, three experts on pedagogy, design for older people and/or interaction design were invited to independently assess the usability of the course content on their laptops in a full-scale model of an apartment. The setting enabled manipulations of the lighting conditions (daylight mode and night mode, change of luminaires), contextual interviews and video observation to identify any problems when participants experimented with the test kit included in the course material. They participated on three occasions lasting 2 h each. Six healthy adults (aged 70+) participated in a similar usability trial in a second round. **Findings**: Experts’ average usability score was 78.3, indicating ‘Good’ usability. However, the interviews did reveal some issues (e.g., difficult or inconsistent terms, unclear instructions). Results were used to refine the course before the second usability trial with six participants. Based on the interviews and usability ratings, the participants were positive about the course, and the instructions were easy to follow. All six participants rated the overall user-friendliness of the course as 6 out of 7. The average usability score was 86.7, indicating ‘Excellent’ usability. Based on the participants’ feedback and interactions in the apartment, changes to the course content included, e.g., clarifying terms, the different types of text links and instructions. Unexpected issues with online enrolment in the course appeared before the second trial because standard instructions developed by the university were not tailored to the participants. **Conclusions**: A two-step usability evaluation by experts in the first round and target users in the second proved valuable. It enabled refinement of the course content and significantly reduced the number of identified usability issues in the second trial. A learning management system seems promising for use in behavior-change interventions. However, the time-limited lab trials restricted a complete evaluation. Therefore, the next step is to pilot the course and evaluate the feasibility in real-world homes.

**Keywords:** behavior-change intervention; usability

### 2.30. Ultra-High-Field MRI Indications That Exposure to Blue-Enriched Light Increases Attention Brain Responses during an Oddball Task

Fermin Balda ^1^, Islay Campbell ^1^, Ekaterina Koshmanova ^1^, Nasrin Mortazavi ^1^, Elise Beckers ^1^, Roya Sharifpour ^1^, Ilenia Paparella ^1^, Alexandre Berger ^2^, Siya Sherif ^1^, Laurent Lamalle ^1^, Christophe Philips ^1^, Pierre Maquet ^1^ and Gilles Vandewalle ^1^

^1^  Sleep and Chronobiology Lab, GIGA-Institute, CRC-In Vivo Imaging Unit, University of Liège, Liège, Belgium
^2^  Institute of Neuroscience (IoNS), Université Catholique de Louvain (UCLouvain), Louvain, Belgium

**Abstract: Introduction**: With the discovery of a new, non-rod, non-cone photoreceptor, new research shows that natural and artificial light regimes have the potential to weaken and strengthen cognition, attention and perception. These effects are mediated in part by melanopsin-expressing intrinsically photosensitive retinal ganglion cells (ipRGCs) that, in contrast to the classical photopic system that is maximally sensitive to green light (550 nm), are very sensitive to blue light (470–480 nm). These photoreceptors not only stimulate alertness, attention, vitality and cognitive performance, but they also influence our biological clock, sleep, thermoregulation and hormonal processes. Using high-resolution ultra-high field (7T) functional magnetic resonance imaging (fMRI), we characterized the neural correlates of the alerting effect of light by assessing the responses to an auditory oddball task. **Methods**: Twenty healthy young subjects (22.95 year ± 2.1 women) were requested to detect rare (20%) deviant tones (100 Hz) among more frequent (80%) standard (500 Hz) ones by pressing a button with their right index finger. In this task, participants were exposed to 30 s blocks of blue-enriched light (4000 K; 308 melanopic EDI lux) and orange monochromatic light (589 nm; 0.2 melanopic EDI lux) interleaved by ~15 s dark periods. **Results**: As many previous studies have reported, there have been activations of temporal, parietal, thalamus, intraparietal sulcus (IPS) and occipital lobes (uncorrected *p* < 0.001) during oddball tasks. There was increased activation in the left IPS and thalamus, under blue light in comparison to orange light (*p* = 0.001 uncorrected). This is in line with other studies that report increased activation in cortical and subcortical regions related to attention as thalamus and IPS under blue light. **Conclusion**: The data show that UHF 7T MRI is powerfully able to analyze many aspects of light and non-visual impact on the subcortical brain. Future analyses with a larger sample size will be able to confirm these preliminary results. Support: FNRS, ULiège, GIGA Doctoral School for Health Sciences, Fondation Léon Frédéric, LIGHTCAP EU-ETN-MSCA.

**Keywords:** oddball; ultra-high-field MRI (UHF MRI); blue light

### 2.31. Metameric Display Light: Melanopsin-Dependent Effects on Slow Eye Movements

Oliver Stefani ^1^, Isabel Schöllhorn ^1^, Robert Lucas ^2^ and Christian Cajochen ^3^

^1^  Psychiatric Hospital of the University of Basel (UPK), Centre for Chronobiology, Basel, Switzerland
^2^  University of Manchester, Manchester, UK
^3^  Centre for Chronobiology, Psychiatric Hospital of the University of Basel, Basel, Switzerland

**Abstract: Introduction**: Visual displays emit optical radiation at short wavelengths, close to the peak sensitivity of melanopsin. The strength of non-image-forming (NIF) responses to light depend on the melanopic equivalent daylight illumination-mEDI. Thus, here we investigated effects of spectral changes of a display backlight on the incidence of slow eye movements (SEMs), an objective measure for sleepiness in humans. **Methods**: Spectrally different white lights that are perceived as the same white tone are called metamers. We developed a metameric display backlight with 5 LED types (440, 480, 500, 550 and 620 nm). By shifting the peak wavelengths of the primary colors, equal stimulation of the three cone types but different stimulation of melanopsin (high (HM) and low (LM) (factor ‘condition’)) was achieved. Seventy-two healthy male participants were examined two times (HM and LM) under controlled laboratory conditions in a randomized within subject design. Participants were divided into four luminance groups.

- Group 1: Luminance 27 cd/m^2^, HM mEDI 15 lx, LM mEDI 4 lx
- Group 2: Luminance 62 cd/m^2^, HM mEDI 33 lx, LM mEDI 9 lx
- Group 3: Luminance 135 cd/m^2^, HM mEDI 70 lx, LM mEDI 21 lx
- Group 4: Luminance 284 cd/m^2^, HM mEDI 146 lx, LM mEDI 48 lx

The incidence of SEMs, derived from the electrooculography (EOG) were quantified every 30 s during the 3.5 h of light exposure period and averaged across 30 min bins. We analyzed the resulting data using Generalized Linear Mixed Models (PROC GLIMIX) with the factors ‘condition’ and ‘time’. **Results**: Data of 72 volunteers entered statistical analysis. For all groups, the factor ‘time’ was significant (*p* < 0.001). Groups 1, 2 and 4 showed significantly more SEMs during LM than HM (*p* < 0.001), (*p* = 0.015) and (*p* < 0.001) respectively, but not group 3. **Conclusions**: Depending on their mEDI, commonly experienced display luminances affect objective sleepiness as indexed by the occurrence of SEMs. To decrease alerting effects of light before bedtime without changing the perceived white tone, reducing mEDI of electronic displays at typical luminances is a successful approach.

### 2.32. Characterizing the Pupil Response under Different Light Conditions during an fMRI Protocol

Islay Campbell ^1^, Elise Beckers ^1^, Ilenia Paparella ^1^, Roya Sharifpour ^1^, Fermin Balda ^1^, Nasrin Mortazavi ^1^, Puneet Talwar ^1^, Alexandre Berger ^2^, Ekaterina Koshmanova ^1^, Siya Sherif ^1^ and Gilles Vandewalle ^1^

^1^  Sleep and Chronobiology Lab, GIGA-Institute, CRC-In Vivo Imaging Unit, University of Liège, Liège, Belgium
^2^  Institute of Neuroscience (IoNS), Université Catholique de Louvain (UCLouvain), Louvain, Belgium

**Abstract: Background**: Light has many non-image-forming (NIF) functions including impacting pupil constriction and stimulation of cognition and alertness, potentially through an impact on the brainstem locus coeruleus (LC). Pupil size modulation is an accessible measure to access light’s physiological impact. Transient dilation in pupil size in response to sensory inputs is considered to be primarily driven by LC phasic activity. Whether this transient pupil dilation is affected by light NIF effects is not established. We aimed to characterize the pupil response to different melanopic light levels and the transient pupil response during an fMRI protocol. **Methods**: Pupil diameter was recorded continuously in eight healthy subjects (24.1 years old ± 2.9; six women) with an eye-tracking device (Eye Link, SR Research, Ottawa, Canada; sampling rate: 1000 Hz), while they completed an 18 min auditory emotional task during an ultra-high-field 7T fMRI scan. During the task, participants listened to emotional and neutral stimuli whilst exposed to pseudo-random alternating 30 to 40 s blocks of polychromatic, blue-enriched light (63, 155, 308 melanopic EDI lux) and monochromatic orange light (589 nm; 0.2 melanopic EDI lux). Light blocks were separated by a 20 s darkness period. Sustained pupil response was computed as the average pupil size during the entire block of light, while the transient pupil response was computed as the change in the pupil size from before and after the auditory stimulus presentation. Statistics consisted of generalized linear mixed models (GLMMs) seeking effects of light melanopic level on either sustained or transient pupil response, with subjects as random intercept and controlling for age, sex and BMI, as well as emotional condition when considering transient pupil response. **Results**: The GLMM analysis revealed that higher melanopic light level was associated with smaller sustained pupil diameter (F(4, 32) = 31.20, *p* < 0.0001). The impact of the light condition on sustained pupil size did not significantly change over the 18 min protocol (light condition x block repetition interaction: F(16, 277) = 0.13, *p* = 1), suggesting that time-in-protocol and prior light blocks did not influence pupil constriction. The transient pupil response was greater under higher melanopic light levels (F(4, 17.7) = 20.15, *p* < 0.0001). Despite qualitative differences in transient pupil size between the emotional conditions, the GLMM did not yield a difference between neutral and emotional stimulations (F(1, 17.71) = 1.09, *p* = 0.3). Furthermore, transient pupil response was not associated with a significant interaction between light and emotional conditions (F(4, 17.7) = 0.81, *p* = 0.5). **Conclusion**: We report preliminary results suggesting that higher melanopic light level triggers a stronger sustained pupil response that appears to remain stable over an 18 min period including alternating short light and darkness exposures. We also show that despite the sustained pupil constriction induced by prolonged light exposure, transient pupil responses to auditory stimulation (which is presumably driven by LC phasic activity) are increased by light level, and potentially particularly if its blue light content is higher. We aim to add more participants to this analysis and to characterize the LC’s activity to light during an fMRI protocol concomitant to pupil measurement. Support: FNRS, ULiège, Fondation Léon Frédéric, LIGHTCAP EU-ETN-MSCA; 

### 2.33. Therapeutic Potential of Bright Daytime Light Exposure in Alzheimer’s Disease Models

Ashwathi Prithviraj, Robert Lucas and Annette Allen

University of Manchester, Manchester, UK

**Abstract:** Neurodegenerative diseases like Alzheimer’s have been studied extensively for several decades now with a rush to find a cure as the number of people afflicted with dementia and related diseases has been rapidly increasing over the years. While researchers are trying to find a drug to cure this disease, studies are also being carried out for potential therapies to alleviate the symptoms of the disease. One of the most severely affected systems in the AD patient is the circadian clock. Patients suffer from disrupted sleep–wake patterns, restlessness, agitation and depression. Circadian rhythm disruption occurs before the onset of dementia and cognitive loss and is one of the early indicators of the disease. Along with the accumulation of Aβ plaques and tau neurofibrillary tangles within the brain, studies have also found that this neurodegeneration extends to the eye. The development of various types of Aβ and tau tangles within the retina, much before the behavioral changes in patients, allows for disease diagnosis with certainty through retinal analysis. Retinal neurodegeneration with significant loss of melanopsin retinal ganglion cells which innervate the circadian pacemaker in the brain could explain the disruptions in the circadian rhythm. Bright light therapy could potentially ameliorate the disrupted circadian cycle and improve cognition, sleep patterns and depression. This project aims to understand the interactions between AD pathology, circadian rhythm dysfunction and retinal deterioration, and investigate the effect of bright-light treatment in ameliorating these conditions. Triple transgenic 3xTg AD mice were housed under bright vs dim 12:12 LD cycles from 6.5–10 months of age. Every 3 weeks, mice from both light conditions were subjected to OCT retinal scans and 3 behavioral tests to compare the progression of the disease and cognitive performance. Working memory and spatial reference memory were assessed with spontaneous and spatial alternation tests using a Y maze. Novelty recognition was studied using the standard novel object recognition tests (NOR). Activity recording was carried out for individually housed mice under each light condition to analyze circadian parameters. Mice housed under bright light did not show a difference in retinal deterioration compared to those housed under dim light although they did show slightly improved cognitive performance. Mean activity levels did not vary between the two test groups during both day and nighttime. Further experiments are being conducted to assess the changes in AD pathology within the brain and retina due to the light treatment as well as to study whether the light treatment has led to differences in the SCN transcriptome.

**Funding:** The LIGHTCAP project has received funding from the European Union’s Horizon 2020 research and innovation programme under the Marie Skłodowska-Curie grant agreement No. 860613. 

### 2.34. Can Light Affect Functional Brain Connectivity? Investigating the Effects of Daytime Light Exposure with Metameric Light and EEG Measures

Elifnaz Gecer ^1^, Luc Schlangen ^2^, Karin Smolders ^2^ and Yvonne de Kort ^2^

^1^  Eindhoven University of Technology, Eindhoven, The Netherlands
^2^  Industrial Engineering and Innovation Sciences, Eindhoven University of Technology, Eindhoven, The Netherlands

**Abstract: Background**: Light elicits important non-image-forming responses (NIF) in the brain, distinct from perceptual vision. These NIF effects are often linked to ipRGCs and their melanopic photoreception (Provencio et al., 1998). Daytime exposure to bright light indoors can have positive acute effects on performance, alertness, vitality and mood (Huiberts et al., 2016; Kaida et al., 2007; Phipps-Nelson et al., 2003; Rüger et al., 2005; Smolders & De Kort, 2014; Smolders al., 2012). However, literature results are heterogeneous and also vary across the objective measures used to evaluate alerting effects of light during daytime. Therefore, more robust objective markers of alertness are needed (Lok et al., 2018). **Aim**: We designed a neuroimaging study to test novel objective markers for light-induced moderations in attention, arousal and vigilance. These markers were assessed under different metameric light conditions. In particular, we aimed to test whether EEG functional connectivity, i.e., the temporal correlation between spatially remote neurophysiological events (Friston et al., 1993), can distinguish acute alerting effects between light conditions having a similar visual appearance but a large difference in melanopsin activation. We also explore spectral power densities in the alpha, delta and theta bands, and whether the three light conditions differ in autonomic nervous activity, subjective sleepiness and cognitive performance. Moreover, we will investigate whether there are correlations between the EEG (functional connectivity, P300, power densities), subjective assessments, ECG and EOG, and task performance measures. **Methods**: The laboratory study consists of a counterbalanced within-subjects design in which participants were exposed to three different light conditions—(a) dim light (9 lx, 2.5 MEDI, 2141K), (b) low-melanopic condition (212lx, 55 MEDI, 2097K) and (c) high-melanopic condition (211lx, 175 MEDI, 1773K)—during one single experimental session. The sessions started at 10 AM with an hour of preparation in dim light before the first experimental light condition was offered. Each of the three experimental light conditions lasted 30 min and was followed by a 30 min exposure to dim light. The experiment uses the in-house engineered Boschman ColorBox that contains 11 LED types, each with a different spectrum and individually controlled, so as to produce a Ganzfeld-like light exposure. The metameric light conditions were calculated by means of the Silent Substitution Method (Spitschan & Woelders, 2018). EEG-derived metrics related to functional connectivity as well as ERPs and EEG power densities were assessed, in parallel to measures related to autonomic nervous activity (ECG, EOG), subjective sleepiness (KSS) and cognitive performance (PVT, Oddball Task). **Results**: Data of 20 participants have been collected, and we will analyze the data using multilevel models to test differences in the outcome parameters as a function of the three lighting conditions. We hypothesize that the functional connectivity levels at the frontal, parietal and occipital electrodes in the high-melanopic condition will be significantly different as compared to the dim light and low-melanopic condition. This research was performed within the European Training Network LIGHTCAP (project number 860613) under the Marie Skłodowska-Curie actions framework H2020-MSCA-ITN-2019.

**Keywords:** metameric light; EEG; functional connectivity; melanopsin; ipRGC; silent substitution; PVT; KSS; Lightbox

### 2.35. Verification of the Effect of a Novel Lighting Source on Circadian Rhythmicity and Mood of Healthy Volunteers

Kateřina Červená ^1^, Katarína Evansová ^1^, Karolína Janků ^1^, Lenka Maierova ^2^, Zdeňka Bendová ^1^ and Jana Koprivova ^3^

^1^  Sleep and Chronobiology Research Centre, National Institute of Mental Health, Klecany, Czech Republic
^2^  University Centre for Energy Efficient Buildings (UCEEB), Czech Technical University in Prague, Prague, Czech Republic
^3^  Sleep and Chronobiology Research Centre, National Institute of Mental Health, Klecany, Czech Republic 2: 3rd Faculty of Medicine, Charles University, Prague, Czech Republic

**Abstract: Background**: In today’s modern society, people spend most of their time in buildings where they are usually exposed to a mere fraction of daylight. This, combined with artificial lighting in the evening, leads to a decrease in the contrast of the photic signal between day and night, which is essential for proper circadian entrainment. Insufficient exposure to bright daylight has been associated with disrupted synchronization of circadian rhythms and related consequences for health, quality of sleep, mood and overall well-being. This study aimed to explore whether winter morning exposure to electric lighting with unique, daylight-mimicking qualities provided by an experimental phototherapy booth prototype called Sun spa would have a significant effect on mood, sleep quality and circadian rhythmicity of healthy volunteers. **Methods**: The ‘Sun spa’ is a cubic interior pavilion with a side of 2.5 m. The ceiling and half of one side wall serve as the source of the light. It provides a corneal illuminance above 8000 lx, which represents Equivalent Daylight Illuminance >7400 lx, with a CCT of 4500 K and a color-rendering index above 90. The light has a diffuse character and balanced spatial distribution with maximal luminance of 8000 cd/m^2^, which is similar to the natural sky. In this preliminary study, 18 participants were exposed to 30 min experimental lighting inside of the ‘Sun spa’ from 10:00 am to 10:30 am for two consecutive workweeks during the winter months. Participants were monitored by MotionWatch8 actigraphy devices starting 2 weeks prior to the experimental lighting exposure and ending 2 weeks post-exposure. Self-report questionnaires and scales on sleep quality and mood were administered 2 days before the experimental lighting exposure and one day after the experimental lighting exposure. **Results**: During the two-week experimental lighting exposure, the Interdaily Stability of the participants´ circadian rhythmicity was significantly increased compared to the two weeks prior and two weeks post-exposure. Self-report scales and questionnaires on anxiety (STAI), depressive symptoms (BDI) and positive and negative affect (PANAS) revealed no significant differences pre- and post-exposure. **Conclusions**: While the experimental lighting exposure significantly increased the interdaily stability of the participants´ circadian rhythmicity during the two weeks of exposure, the intervention had no significant effect on self-report ‘mood’ correlates. However, part of the experiment, affecting a subset of participants, took place when the war had already started and participants reported it affected their sleep quality and emotions. Moreover, it remains to be determined to what extent the increased interdaily stability actually reflected the required commitment to attend the ‘Sun spa’ regularly.

**Keywords:** phototherapy; full-spectrum light source; daylight-mimicking light source; actigraphy; circadian rhythmicity; mood

**Funding:** The Technology Agency of the Czech Republic grant no. FW02020025 (Stable and mobile devices to support circadian synchronization, treatment and prevention of mental disorders through full-spectrum light phototherapy).

### 2.36. Quantification of Light Exposure Characteristics Modulating Non-Visual Responses with Light Dosimetry

Steffen Hartmeyer ^1^, Frederic Rudawski ^2^, Martine Knoop ^2^ and Marilyne Andersen ^1^

^1^  Laboratory of Integrated Performance in Design (LIPID), École Polytechnique Fédérale de Lausanne (EPFL), Lausanne, Switzerland
^2^  Fachgebiet Lichttechnik, TU Berlin, Berlin, Germany

**Abstract:** (1) Background: Extensive laboratory research has shown that physiological and behavioral responses to light are modulated by different light exposure characteristics, that is, spectral composition, intensity, duration, timing, temporal dynamics, prior history and spatial distribution. However, it is unclear to what extent these laboratory-derived relationships generalize to complex real-world personal light exposure (PLE). To provide a basis for addressing this question, we conducted a comprehensive review of previously published dosimetry studies to identify methods and metrics for quantifying light-exposure pattern characteristics. Since no dosimetry study has been found that quantified spatial distribution in PLE, we additionally conducted an exploratory pilot study of spatiotemporal dynamics of PLE to assess the potential relevance of spatial distribution of light in real life. (2) Methods: In the pilot study, person-bound exposure time-series of melanopic radiance distribution and spectral irradiance were collected in different everyday environments. Measurements were collected using a novel setup, consisting of wide-angle video-photometers and spectrally resolved dosimeters worn at the chest and the forehead. Light distribution was measured at a high temporal resolution, with an epoch of 2 s at the chest and 10 s at chest and forehead. (3) Results: Analyses of the spatially resolved measurements show that spatial variability across the visual field can differ substantially between different everyday environments and contexts: spatial variability tended to be higher in indoor environments and environments dominated by electric lighting, and spatiotemporal variability was generally higher for measurements at the forehead than at the chest. Furthermore, hypothetical modelling of retinal spatial sensitivity suggests that the impact of spatial distribution on non-visual responses may strongly depend on the context and is likely to be small. (4) Conclusions: The pilot study is the first to provide spatially resolved measurements of PLE, showing that it is possible to quantify spatial distribution in dosimetry. The collected data may enable many interesting opportunities to examine and model retinal spatial sensitivity at the level of individual ganglion cells up to quantifying the effective light stimulus for non-visual responses. Finally, this study complements our comprehensive review, which provides a wide range of metrics to quantify different light-exposure pattern characteristics. Taken together, these efforts form a basis towards a consensus framework for light-dosimetry studies and ultimately contribute to fostering the efficacy of research in real life to answer pressing questions in many applied contexts, be it architecture, shift work or personal lifestyle.

**Keywords:** non-visual; circadian; lighting; dosimetry; field studies; quantification; spatial distribution

**Funding:** This research is part of the LIGHTCAP project, that has received funding from the European Union’s Horizon 2020 research and innovation programme under the Marie Sklodowska-Curie Innovative Training Networks (ITN) grant agreement No. 860613.

### 2.37. Changes in Circadian Rhythmicity during COVID-19 Pandemic Lockdowns

Katarína Evansová ^1,2^, Anna Sochůrková ^1,3^, Jakub Březina ^1,3^, Kateřina Červená ^1,4^, Ondřej Novák ^1^ and Jana Koprivova ^1,2^

^1^  Sleep and Chronobiology Research Centre, National Institute of Mental Health, Klecany, Czech Republic
^2^  Faculty of Medicine, Charles University, Prague, Czech Republic
^3^  Department of Psychology, Faculty of Arts, Charles University, Prague, Czech Republic
^4^  Department of Physiology, Faculty of Science, Charles University, Prague, Czech Republic

**Abstract: Background**: For many people, the COVID-19 pandemic was an intrusion into their daily activities and their work and personal lives. Due to this pandemic, people were forced to spend their time mainly in their homes, which significantly reduced their activities. These restrictions have had a major impact on people, both in terms of their physical and mental conditions. Thanks to this exceptional situation, we have a unique opportunity to examine changes in sleep in the natural environment after removing social influences, such as a strict work schedule. At the same time, in addition to contributing to the development of knowledge about the functioning of people in domestic isolation, this research also has possible applicable potential (prevention of health problems associated with desynchronization of biological and social rhythm, streamlining sleep by adjusting working hours to individual time preferences, etc.). In this study, we ask whether in different chronotypes there will be a change in the rhythm of sleep and wakefulness, as well as in the quality of sleep, during home isolation. **Methods**: The study included 38 participants (including 4 people who were discarded) of extreme chronotype monitored by actigraphy and examined for anamnesis, mood and sleep patterns using online questionnaires. Participants were monitored using MotionWatch8 actigraphy devices for the duration of the pandemic emergency. Changes in circadian rhythms were also monitored in 104 participants and in this case only using online questionnaires, especially a sleep diary, which the participants filled in every day using the application during the spring lockdown. The following questionnaires were used in these studies MCTQ, STAI, PSQI, BDI and PANAS. **Results**: Preliminary results reveal no significant changes in the MCTQ questionnaire-based marker of the circadian phase during pandemic lockdown restrictions compared to the pre-pandemic baseline. However, actigraphy results show increased intradaily variability in morning chronotypes compared to evening and intermediate chronotypes. In the self-report questionnaire assessing sleep quality (PSQI), there was a significant improvement in sleep quality during the lockdown, but only in the group of intermediate chronotypes. **Conclusions**: Contrary to our expectations, the circadian phase during lockdown restrictions did not change significantly compared to baseline in any chronotype group. However, though the results are still preliminary, the increased intradaily variability in morning chronotypes over evening and intermediate types is an interesting finding that might be explained by reduced ability to adapt to the lack of social schedule in morning types compared to evening and intermediate types.

**Keywords:** sleep during COVID-19; actigraph; circadian rhythms

**Funding:** The Technology Agency of the Czech Republic grant no. FW02020025 (Stable and mobile devices to support circadian synchronization, treatment and prevention of mental disorders through full-spectrum light phototherapy). Cooperation, Neuroscience, Charles University.

### 2.38. How Do SCN Neurons Read the Time of the Day in Diurnal and Nocturnal Rodents

Patrycja Orlowska-Feuer, Beatriz Bano-Otalora and Robert Lucas

University of Manchester, Manchester, UK

**Abstract:** The master circadian clock in the suprachiasmatic nucleus (SCN) of the hypothalamus detects day/night changes in environmental light intensity and uses this information to appropriately coordinate daily rhythms in metabolism, physiology and behavior. Previous electrophysiological studies have shown that the majority of SCN neurons respond to light steps with a maintained increase in firing, the magnitude of which is defined by stimulus irradiance. That type of ‘irradiance response’ is considered to reflect the SCN’s use of retinal input to track the time of the day. However, most of those studies were performed on nocturnal rodents and the extent to which such responses are retained in day-active animals is less unclear. Here, we set out to provide a detailed comparison of hypothalamic light responses between a diurnal murid rodent, Rhabdomys pumilio, and nocturnal mice by performing in vivo acute multielectrode recordings. The visual response properties of SCN cells were overall very similar between species, with the majority of neurons excited by light stimulation and responding in either a sustained or a transient manner. R. pumilio had smaller response latencies, higher baseline firing rate activity and were more sensitive to light. In both species, sustained neurons tracked changes in irradiance presented either as simple light steps or when more naturalistic stimuli (such as temporal white noise or chirp) were superimposed. A strong species difference was observed in the peri-SCN region though, with visual responses much more common in R. pumilio (appearing in 70% of neurons vs. 10% in mice). These peri-SCN responses were not reliably irradiance coding, but biased towards more transient responses to light increments and/or decrements. Our findings show that regardless of the temporal niche, many SCN neurons code irradiance in their maintained firing rate. On the other hand, the high percentage and sensory diversity of light-responsive neurons outside the SCN in R. pumilio suggest widespread processing of more sophisticated visual information in the peri-SCN region of these diurnal animals.

**Funding:** Marie Sklodowska-Curie Actions Individual Fellowships (897951), Polish National Agency for Academic Exchange, Bekker Programme (PPN/BEK/2018/1/00192); Biotechnology and Biological Sciences Research Council (BB/P009182/1).

### 2.39. Effects of Temporal Light Modulation on Workers’ Cognitive Performance, Mental Workload and Well-Being

Andreas Wojtysiak

Federal Institute for Occupational Safety and Health, Dortmund, Germany

**Abstract:** The extensive transition from traditional light sources to light-emitting diodes (LEDs) facilitates the use of new functions in the lighting of workplaces, such as dimming, optical data transmission and circadian daylight simulation. These functions often come with light modulations, usually referred to as visible or invisible flicker, the effect of which on humans is not yet sufficiently understood. It is widely recognized that the existing occupational health and safety regulations protect employees against known hazards from light modulations like perceptible flickering. In the area of more subtle influences on visual processes and activities that require constant high concentration for sustained periods; however, there are still gaps in knowledge. Especially for the frequent use of the so-called PWM (pulse width modulation) method for light dimming and for new light-based data transmission technologies, the current body of evidence does not appear to be convincingly robust. Scientific studies indicate that even with fast and therefore invisible flickering, light modulation may represent a load on the exposed person because it is still registered in the sensory and nervous system. Visual perception and mental performance can be altered and increased stress may be an outcome. The poster will present objectives and planned methods of a beginning project in the Federal Institute for Occupational Safety and Health in Germany. This project aims to expand the knowledge base for the safe use of dimmable and data-controlled lighting in the workplace. It will investigate the effect of light modulations on cognitive performance, mental workload and the well-being of employees in a laboratory subject study with established subjective and objective tests as well as modern neurophysiological methods. 

### 2.40. Usability and Acceptability of Corneal-Plane α-Opic Dosimetry in a 24 h Field Trial

Manuel Spitschan ^1^, Sophie Garcia ^2^, Eljoh Balajadia ^2^, Janine Stampfli ^3^ and Björn Schrader ^3^

^1^  TUM Department of Sport and Health Sciences (TUM SG), Technical University of Munich
^2^  Department of Experimental Psychology, University of Oxford, Oxford, UK
^3^  Lucerne School of Engineering and Architecture, Lucerne, Switzerland

**Abstract: Background**: Ocular light exposure influences our human physiology and behavior. Recently, an international expert group published recommendations (Brown et al., PLoS Biology, DOI: 10.1371/journal.pbio.3001571) for criterion light levels during daytime, evening and nighttime that support these non-visual influences. However, it is currently unknown whether these criterion light-exposure levels are met in practice, necessitating wearable dosimeters. Here, we evaluated the use of a novel spectacle-mounted corneal-plane light dosimeter (Stampfli et al., CIE Proceedings, DOI: 10.25039/x48.2021.op18; https://light-dosimeter.ch/ accessed on 31 August 2022) to measure ocular light exposure. **Methods**: Eighteen (n = 18) full-time students (20.1 ± 1.6 years, 9 female) living in the Oxford Ring Road wore a light dosimeter-measuring photopic illuminance, CCT, α-opic irradiance and α-opic equivalent daylight illuminance (EDI) following CIE S 026/E:2018 as well as device tilt for a period of approximately 24 h in an unconstrained ecological setting. After the 24 h measurement period, participants completed Likert-scale questionnaires probing social, usability and intrinsic motivation. Additionally, we asked for open-ended feedback and comments, which we subjected to a thematic analysis. **Results**: Ocular light exposure profiles could be readily measured with the corneal-plane light dosimeter, producing distinct temporal light exposure patterns that varied between different individuals. Participants rated wearing the device as acceptable and usable. The thematic analysis revealed two main themes that participants were concerned with: size, weight and stability of the device, and positive and negative reactions from other people. **Conclusion**: Our study indicates that corneal-plane dosimetry may be feasible for measuring ocular light exposure in the field, leading to novel insights into the relationship between light exposure and physiological outcomes. The study highlights that for long-term use and convenience, miniaturization of sensors for use in the corneal plane may be necessary.

### 2.41. Development of the ENLIGHT Reporting Guidelines for Human Laboratory-Based Light Exposure Interventions

Manuel Spitschan ^1^, Raymond Najjar ^2^, Elise McGlashan ^3^, Renske Lok ^4^ and Laura Kervezee ^5^

^1^  TUM Department of Sport and Health Sciences (TUM SG), Technical University of Munich, Munich, Germany
^2^  Department of Ophthalmology, Yong Loo Lin School of Medicine, National University of Singapore, Singapore
^3^  School of Psychological Sciences and Turner Institute for Brain and Mental Health, Monash University, Selangor, Malaysia
^4^  Center for Sleep Sciences and Medicine, Stanford University, Stanford, CA, USA
^5^  Group of Neurophysiology, Department of Cell and Chemical Biology, Leiden University Medical Center, Leiden, The Netherlands

**Abstract: Background**: The wide-reaching effects of light on human health and wellbeing have been highlighted by various basic laboratory findings. However, there is no consensus or standard on how light characteristics in these studies should be reported. The objective of the ENLIGHT project is to develop the first consensus-based reporting guidelines for laboratory-based light-exposure interventions. **Methods**: The project follows a modified Delphi process with four rounds. Rounds 1 and 2 serve to identify the initial set of items for the reporting guidelines using importance ratings, leading to an initial draft of a checklist. Feedback on the checklist will be discussed in synchronous discussions in Round 3, with a final questionnaire-based query in Round 4. The project is registered on the EQUATOR Network and the Open Science Framework. We are currently completing Round 2. **Results**: In Round 1, we invited 115 experts, of whom 65 (36 female) completed Round 1. Participants were mainly based in Europe and North America, with representation from Asia, Australia and South America. More than 90% reported having a doctoral degree (median award year 2004, with range 1968–2021), and the majority (75.4%) reported being a principal investigator or equivalent. From an initial item pool of 61 items, 24 reached the threshold for definitive inclusion as they were rated important by ≥75% of participants. The 37 items that did not reach this threshold were rated as either unimportant, or unknown, by between 8 and 60% of participants. Hence, no items were rated as unimportant by ≥75% of participants, so none of the items met the threshold for definite exclusion. For 18 items, ≥20% of participants reported not recognizing the item and consequently not being able to evaluate its importance. These items mostly concerned color (rendition) metrics as well as CIE S026 α-opic quantities. We constructed a draft checklist from the Round 1 results, which is currently being evaluated in Round 2. **Conclusion**: The ENLIGHT project is still ongoing, with the goal of synchronous discussion sessions (Round 3) taking place in June/July and the final questionnaire-based round (Round 4) being completed in Fall 2022.

### 2.42. Metameric Display Light: Melanopsin-Dependent Effects on Sleep Latency, Melatonin and Visual Comfort

Isabel Schöllhorn ^1^, Oliver Stefani ^1^, Robert Lucas ^2^, Manuel Spitschan ^3^ and Christian Cajochen ^3^

^1^  Centre for Chronobiology, Psychiatric Hospital of the University of Basel, Basel, Switzerland
^2^  Centre for Biological Timing, School of Biology, Faculty of Biology Medicine and Health, University of Manchester, Manchester, UK
^3^  TUM Department of Sport and Health Sciences (TUM SG), Technical University of Munich, Munich, Germany

**Abstract: Background**: Evening exposure to display light has been shown to prolong sleep latency and to have phase-shifting effects on circadian melatonin rhythms. All three photoreceptors (cones, rods and melanopsin-containing ganglion cells) contribute to the so-called non-image-forming (NIF) effects of light. To better understand isolated melanopsin-dependent NIF effects of evening display light, we generated light settings that matched cone excitation (metamers), but differed maximally in melanopsin contrast. Here, we aimed to observe melanopsin-dependent effects on sleep latency, melatonin concentration and melatonin onset as well as visual comfort in different luminance levels. **Methods**: Seventy-two healthy, male participants completed a 2-week study protocol. Volunteers were assigned to one of four groups, which differed in luminance levels (27 cd/m^2^-285 cd/m^2^). Within the four groups, each volunteer was exposed to a low melanopic (LM) and high melanopic (HM) condition. The metameric LM and HM differed in melanopic equivalent daylight illumination (mEDI): Group 1: mEDI 4 lx vs. mEDI 15 lx, Group 2: mEDI 9 lx vs. mEDI 33 lx, Group 3: mEDI 21 lx vs. mEDI 70 lx, Group 4: mEDI 48 lx vs. mEDI 146 lx. The two 17 h study protocols comprised 3.5 h of light exposure, starting 4 h before habitual bedtime, 8 h of bedtime and one hour of morning dim light. Polysomnographically assessed sleep latency (time between lights off and first occurrence of sleep stage N2) was manually scored according to AASM. Before, during and after light exposure, salivary melatonin levels were measured in half-hourly intervals throughout scheduled wakefulness. To evaluate visual comfort, the volunteers filled in a Visual Comfort Questionnaire (e.g., acceptance of light situation, brightness, light color, and glare perception). **Results**: Sleep latency was significantly longer after HM than LM in Group 4 (*p* = 0.02). During HM, melatonin concentrations were significantly reduced in all groups compared to LM (Group 1: *p* < 0.01, Group 2: *p* = 0.02, Group 3: *p* < 0.01, Group 4: *p* < 0.02). Morning melatonin concentrations were significantly higher after HM in Groups 1 and 4 than after LM (*p* = 0.02, *p* < 0.01). Additionally, HM delayed melatonin onsets in Groups 1, 3 and 4 (*p* < 0.001, *p* = 0.02, *p* = 0.001). Group 3 preferred the light color during HM compared to LM (*p* < 0.01). There were no further significant light effects on visual comfort. **Conclusions**: We have first evidence for an isolated melanopsin-dependent impact of evening display light prolonging sleep latency and delaying melatonin onset. Furthermore, relatively low melanopic irradiances of evening display light elicited NIF effects in the evening and morning. Therefore, since many people are exposed to display light in the evening and at night, reducing melanopic radiance may be a crucial key to reducing unwanted NIF effects of light. Using metameric display light allows manipulating melanopsin excitation independent of visual appearance.

### 2.43. Sleep Education in the Elderly

Katarína Evansová ^1,2^, Lucie Urbanová ^1,3^, Ondřej Novák ^1^, Kateřina Červe ^1,4^, Aleš Bartoš ^1^

^1^  Sleep and Chronobiology Research Centre, National Institute of Mental Health, Klecany, Czech Republic
^2^  3rd Faculty of Medicine, Charles University, Prague, Czech Republic
^3^  1st Faculty of Medicine, Charles University, Prague, Czech Republic
^4^  Faculty of Science, Charles University, Prague, Czech Republic

**Abstract: Background**: Insufficient and poor quality sleep can cause a number of problems, including lowering immunity, worsening mood or exacerbating already existing health complications, which can fundamentally affect vulnerable individuals such as the elderly. Sleep is one of the pillars of health and it is very easy to disrupt the sleep cycle. Although sleep is important in assessing the overall condition of patients, sleep problems in the elderly are not given enough attention. Aging is associated with a phase shift of circadian rhythm and sleep disorders in the elderly are very common (75 years old = 40 +%). These disorders adversely affect behavior and sleep quality, especially in the elderly, and are also closely linked to cognitive function and experience. The main goal of the study is to determine the effect of sleep and light education on sleep, anxiety and depressive symptoms in the elderly. **Methods**: Thirty-two participants took part in a four-week sleep education program. SE took place once a week for 60–90 min during which the participants were instructed in sleep and light hygiene, sleep processes and practiced relaxation techniques. Participants completed weekly questionnaires before each meeting and wore actigraphs for 6 weeks (2 before and 4 during education), filled out sleep diaries, and wore light-blocking glasses 120 min before bedtime. **Results**: The research results showed a significant difference before and after sleep education in both objective actigraphy measures (Motion Watch), as well as subjective (self-report questionnaires). The sleep education program led to increased subjective quality of sleep (PSQI), decreased sleep latency (MW), as well as depressive symptoms (BDI), anxiety (STAI X1) and sleepiness (ESS). **Conclusions**: Sleep education has proven to be an effective method to reduce sleep problems and the occurrence of depressive and anxiety symptoms, which in combination with other methods achieves more effective results. In our program combining sleep and light education, using the rules of sleep and light hygiene, relaxation exercises and stimulus control, we achieved significant results both in the subjective evaluation of participants and in objective actigraph measurements.

**Keywords:** sleep and light hygiene; education; sleep in the elderly; actigraph; depression; anxiety

**Funding:** Czech Czech Health Research Council grant no. NV18-07-00272 (Sleep and brain changes related to memory decline in mild cognitive impairment), Cooperatio, Neuroscience, Charles University.

### 2.44. Light Exposure of Indoor and Outdoor Workers in Different Occupations

Ljiljana Udovicic and Corina Varga

Federal Institute for Occupational Safety and Health, Dortmund, Germany

**Abstract: Background**: Light exposure at night during night shift work, as well as low daytime light levels caused by predominantly indoor activities and too little time spent outdoors can contribute to the desynchronization of endogenous circadian processes in the human body. In an ongoing field study, we are evaluating and comparing the light exposure of different occupations: indoor employees who work night shifts and are therefore exposed to light at night, daytime employees who work indoors and are supposed to experience low light exposure during the day, and outdoor working employees. **Methods**: The participants in the field study measured personal light exposure continuously over one working week in two different seasons (winter and spring/summer). The data contain 24 h light exposure from natural and artificial light sources. The light exposure was assessed using calibrated actimetry ActTrust devices (Condor Instruments), placed on the outer layer of the clothing at chest level. The interpretation of the personal light exposure data was supported by an activity diary of the participants. **Results**: In our study, we evaluated 24 h light exposure of the following occupations:

- indoor employees working night shifts—elderly care nurses, order pickers;
- daytime working employees working indoors—hotel staff, software developers;
- outdoor employees—gardeners, waste collectors.

**Keywords:** personal light exposure; workers; indoor; outdoor; occupation

The light exposure shows the effects of selected times of day, seasons (winter, spring/summer) and locations (indoors, outdoors). On working days, it is essentially determined by the working time and reaches its maximum on the way to and from the workplace, as long as these commuting times include exposure to daylight. Indoor daytime working employees experience the highest light exposures after work (on the way home, while doing shopping, etc.) and in the afternoon (leisure activities). The results will be presented in terms of illuminance, luminous exposure (illuminance × exposure duration) and an exposure time above certain illuminance thresholds for the named occupations. **Conclusion**: Light exposure of night shift working employees is very low compared with those of outdoor workers, especially in the case of employees who work 10 h night shifts. Night shift workers sleep during the day and invariably can experience only very short periods of daylight, in particular in winter. Light exposure of indoor daytime working employees is higher, but compared with the light exposure of outdoor workers, these employees also experience low light exposure during the day.

### 2.45. Impact of Integrative Lighting on Seniors

Lenka Maierova ^1^, Eva Andrlikova ^2^, Katerina Skalova ^2^, Jana Koprivova ^3^ and Zdeňka Bendová ^2^

^1^  University Centre for Energy Efficient Buildings (UCEEB), Czech Technical University in Prague, Prague, Czech Republic
^2^  Sleep and Chronobiology Research Centre, National Institute of Mental Health, Klecany, Czech Republic
^3^  Faculty of Medicine, Charles University, Prague, Czech Republic

**Abstract:** People in modern society spend typically 90% of their time indoors and in population groups such as seniors, this time increases to almost 100%. To compensate for the well-described negative aspects of limited access to daylight, lighting systems installed in senior facilities need to be developed as ‘integrative lighting’, i.e., their light must integrate information for both visual system and non-image-forming light perception. We developed an integrative lighting system for senior residency in Beroun (CZ), particularly in the clients’ apartments, main community dining room, nurses’ room and handicraft workshop. A key attribute of new lighting is that it enhances spectral and intensity differences between day and night. According to the time of the day, light gradually adapts its intensity, spectral and spatial distribution in order to mimic daylight. During the nighttime, an independent system of dim, amber light was installed to assist residents with safe movement around the apartment without suppressing their melatonin production. To monitor the lighting conditions, we measured the corneal illuminance of a person on the bed and at the table and evaluated the visual comfort by using luminance analyses and glare rating calculations. We controlled the overall energy consumption of the lighting system. Further, we investigated the effects of the integrative lighting system on the clients as well as on the employees in two periods, i.e., before (in November) and 3 months after the installation of the lighting system (in February), we determined the melatonin profile and activity/rest cycle using actigraphy. We assessed seniors’ subjective ratings in questionnaires and cognitive tasks. Our data show a significant improvement in interdaily stability of the circadian rhythm in the activity–rest cycle measured by actigraphy in clients after 3 months of living in the new lighting system. In addition, integrative lighting improved the amplitude of the circadian rhythm in salivary melatonin, mainly due to the suppression of daytime melatonin levels. To control for the influence of season, melatonin levels were also determined in employees, with no difference between collections in November and February. Unfortunately, the questionnaires assessing sleep quality, mood, self-care and cognitive performance were not evaluable due to the COVID-19 pandemic. Nevertheless, most clients commented positively on the change in lighting conditions in their rooms and other areas of the senior residence. In particular, night lighting was very highly appreciated for its simple (automatic) control and even distribution of soft dim light, which allows seniors better orientation and safe movement in space. Our findings suggest high visual comfort, positive impact on circadian rhythms and general wellbeing, and positive user acceptance of the new lighting while close to zero increase of lighting energy.

### 2.46. Chronotypes in the Age of COVID-19: Associations with Social Rhythms and Psychological Wellbeing over Time

Lisa M. Wu and Ali Amidi

Aarhus University, Aarhus, Denmark

**Abstract: Background**: A body of evidence suggests that one’s chronotype is related to psychological well-being, with evening chronotypes being more susceptible to poorer psychological well-being. Evening chronotypes are also particularly prone to social jetlag in which there is a discrepancy between social and biological time. Social rhythms reflect the regularity of a person’s daily life activities, including when a person gets out of bed, starts work and has dinner. Research also indicates that social rhythms may have implications for a person’s psychological well-being, including depressed mood, sleep and anxiety. Thus, evening types may be more prone to the negative effects of poor social rhythmicity upon psychological well-being. With the arrival of COVID-19, much of the world entered lockdowns between March and May 2020 requiring or encouraging individuals, for the most part, to conduct their daily life activities from home, thus potentially providing evening types the opportunity to conduct activity according to biological time. In this study, we examined whether morning and evening chronotypes experienced different trajectories of psychological well-being and social rhythmicity in the early months of the COVID-19 pandemic. **Methods**: Seventy-three participants were recruited from the community through social media networks. Individuals who gave consent to participate completed surveys for 7 days, at each of three time points, on a monthly basis. Participants were assessed on measures of psychological well-being (i.e., depressed mood, anxiety, perceived stress, sleep quality, fatigue, social isolation, cognitive ability and general well-being). Linear mixed models were undertaken to investigate associations between chronotype (morning vs. evening) and markers of psychological well-being over time. In addition, we modified the Social Rhythm Metric-5 to create a social rhythmicity score based on a comparison with self-reported habitual timing of activities pre-COVID-19. We then used a linear mixed model to examine the association between chronotype and social rhythmicity. **Results**: Linear mixed models indicated that evening types experienced significantly higher levels of anxiety (*p* = 0.04), and lower general well-being over time than morning types (*p* = 0.003). There was also a trend indicating that evening types experienced greater fatigue over time than morning types (*p* = 0.08). Finally, evening chronotypes experienced significantly poorer social rhythmicity over time (*p* = 0.047). Conclusions: Findings from this study undertaken during the early months of the COVID-19 pandemic corroborate research indicating that evening types are more susceptible to poorer psychological functioning and poorer social rhythmicity despite the fact that the majority of participants in this study were in the midst of pandemic lockdowns.

**Keywords:** social rhythm metric; circadian rhythms; COVID-19; psychological well-being; chronotype

**Funding:** European Union’s Horizon 2020 Research and Innovation Programme under the Marie Sklodowska-Curie Program 754513 and the Aarhus University Research Foundation.

### 2.47. A Chronodisruptive Light/Dark Cycle Impairs the Central Regulation of Female Fertility

Valerie Simonneaux

CNRS & University of Strasbourg, Strasbourg, France

**Abstract:** In female mammals, cycles in reproductive function depend on both a biological clock synchronized to the light/dark cycle, and a balance between the negative and positive feedbacks of estradiol which concentration varies during ovary maturation. In women, studies report that chronodisruptive environments, notably those experienced in shiftwork conditions, may impair fertility and gestational success. We explored in female mice how shifted light/dark cycles may alter the hypothalamo–pituitary–ovarian axis. Mice exposed to a single 10 h phase shift exhibit a moderate and transient alteration of the LH surge timing and estrous cyclicity. By contrast, mice exposed to a chronic shift (successive rotations of 10 h phase advance for 3 days followed by 10 h phase delay for 4 days) exhibited a severely altered reproductive activity, with an impaired kisspeptin regulation of the preovulatory LH surge, disrupted estrous cycles and a significant reduction in gestational success. 

### 2.48. Melatonin: How to Tell and Change the Time of the Circadian Clock

Vikki Revell

University of Surrey, Surrey, UK

**Abstract:** The hormone melatonin is secreted from the pineal gland in a rhythmic fashion driven by the master circadian clock in the suprachiasmatic nuclei (SCN). Typically, in humans, the onset of melatonin production begins a few hours before sleep with levels peaking during the night and then returning to basal levels around waking. The endogenous melatonin rhythm is recognized as a reliable marker of the human circadian clock which can be used not only to understand the timing of an individual’s endogenous clock but also to assess the impact of circadian targeted interventions. Exogenous melatonin, in supraphysiological doses, is administered for both its soporific effects and its ability to shift the timing of (phase shift) the circadian clock. Phase response curves (PRCs), describing the relationship between the timing of administration of melatonin and the resultant phase shift, have been generated for different melatonin doses. This talk will explain the physiology and regulation of endogenous melatonin, as well as how to measure levels of melatonin, and its metabolites, in different matrices. In describing methodology for the laboratory and real world, consideration will be given to factors that influence melatonin levels (e.g., environmental light, posture) which should be taken into account when designing protocols. Finally, PRCs to different doses of melatonin will be described in the context of their potential clinical applications.

**Keywords:** melatonin; circadian; phase response curve, dose

### 2.49. Circadian Regulation of Neuroimmune Function: Implications for Cognition and Behavior

Louise Ince, Ruizhuo Chen, Jeffrey Darling, Lourdes Davis, Emily Greenough, Aidan Weitzner, Akshay Prabhakar, Anusha Dabak, Krishi Manem, Andrew Gaudet and Laura Fonken

University of Texas at Austin, Austin, TX, USA

**Abstract:** Neuroinflammation can be induced by signals originating both inside and outside the central nervous system (CNS). Exposure to pathogens or injury activates immune cells to initiate inflammatory cascades, which are communicated to the CNS through various pathways, including neural and blood-borne routes. Inflammatory signals are propagated within the brain by resident CNS glia (microglia and astrocytes), endothelial cells and peripherally derived immune cells. These inflammatory signals trigger cellular and physiologic changes which manifest as sickness behavior, e.g., social withdrawal, cognitive impairment and anhedonia. Sickness behaviors are adaptive in the short term and facilitate recovery; however, pathways set up to confer the sickness response can be hijacked in other conditions with chronic inflammation, leading to pathological behavioral changes. The circadian clock is a critical regulator of inflammatory processes, both in the periphery and within the CNS. We therefore sought to assess the influence of biological rhythms upon periphery-CNS immune crosstalk and the consequences on cognitive and mood-related behaviors. We hypothesized that there are diurnal rhythms in neuroimmune function, and that disrupted circadian function, via (a) cell-specific manipulation of the molecular clock, (b) during aging or (c) after exposure to dim light at night (dLAN), leads to altered inflammatory signaling and behavioral changes. First, we investigated whether an intact microglia clock is essential for optimal neuroimmune regulation by performing behavioral testing and inflammatory challenges in mice with microglia-specific knockout of the core clock gene Bmal1 (Bmal1flox/floxTmem119CreERT2, microglia-Bmal1−/−). In Bmal1-proficient mice, lipopolysaccharide (LPS, 0.5 mg/kg i.p.) injection during the mid-light (vs mid-dark) phase elicits enhanced inflammatory cytokine production in the hippocampus. In contrast, microglia-Bmal1−/− animals no longer showed time-of-day variation due to an increased inflammatory response during the dark phase. Enhanced inflammation was also seen in aged wild-type mice (18 months), which showed increased baseline inflammatory cytokine production during the dark phase relative to young mice (3 months). Consistent with inflammation-associated behavioral changes, aged mice also showed decreased sociability. However, both nocturnal inflammation and social withdrawal were reduced using a time-restricted feeding protocol in aged mice, suggesting that manipulation of circadian rhythms may decrease age-associated neuroinflammation and improve social behavior. In addition, we found that circadian disruption in adolescent mice via exposure to dim light at night (dLAN) (12:12 light (150 lx)/dim (15 lx) cycle) increases depressive-like behavior in adulthood and exacerbates LPS-induced neuroinflammation in a sex-specific manner, further highlighting the influence of circadian function on mood and inflammatory signaling. Our results indicate that circadian disruption can worsen depressive- and anxiety-like behaviors, via potentiation of neuroinflammatory pathways, and suggest that strategies to reinstate or amplify dampened circadian rhythms (e.g., in aging) could reduce neuroinflammation and improve mood. Grant support: Research supported by faculty start-up funds to LKF and NIH R01 (R01AG062716) to LKF.

**Keywords:** microglia; behavior; mood; inflammation

### 2.50. Antidepressant Chronotherapeutics Normalizes Prefrontal 1H-MRS Glutamate in Bipolar Depression

Elisa M.T. Melloni ^1^, Beatrice Brav ^1^, Sara Poletti ^1^, Sara Dallaspezia ^2^, Barbara Barbini ^2^, Raffaella Zanardi ^2^ and Francesco Benedetti ^1^

^1^  University Vita-Salute San Raffaele, Milano (2) Scientific Institute IRCCS Ospedale San Raffaele, Milano, Italy
^2^  Scientific Institute IRCCS Ospedale San Raffaele, Milano, Italy

**Abstract: Background**: Bipolar Disorder (BD) is associated with impaired synaptic function, cellular resilience and plasticity, and dysfunctional glutamatergic neurotransmission has been proposed both as a biological underpinning of mood disorder and as a target for rapid-acting antidepressant treatments. Combined total sleep deprivation and light therapy (TSD+LT) can prompt antidepressant response in drug-resistant bipolar depression. Here we explored the effects of TSD + LT on dorsolateral prefrontal cortex (DLPFC) glutamate and/or glutamine+glutamate (Glx) levels. **Methods**: We studied single voxel 1H-MRS measures of DLPFC Glu and Glx levels of 48 healthy participants and 55 inpatients with a major depressive episode in the course of BD, a subset of which (N = 23) underwent three cycles of repeated TSD+LT and were evaluated before and after treatment. Treatment effects of mood and on Glu and Glx concentrations were analyzed in the context of the Generalized Linear Model (GLM), correcting for age, sex and lithium treatment. **Results**: Higher concentrations of Glu (adjusted Z = −2.189, *p* = 0.0285) and Glx (adjusted Z = −3.13, *p* = 0.0017) were observed in BD patients compared to HC. Treatment caused a significant rapid reduction of depressive symptom severity over time (F = 63.98, *p* < 0.01). Change in depression levels after TSD+LT treatment was significantly influenced by delta change in Glu levels (LR χ2 = 4.619, *p* = 0.0316) and in Glx levels (LR χ2 = 4.486, *p* = 0.0341). **Conclusion**: A reduction in Glu and Glx levels associated with depression could contribute to the mechanism of action of TSD+LT, directly acting on glutamatergic neurons, or to the interaction between the glutamatergic system and dopamine (DA) and serotonin (5-HT) levels, known to be targeted by TSD. This is in line with several studies showing glutamatergic modulation effects of antidepressants and mood stabilizing agents. This finding deepens our understanding of antidepressant effects of chronoterapeutics and strengthens the impact of biomarker research into clinical practice, providing new insights for the development of innovative therapeutic strategies for bipolar disorder.

### 2.51. Three-Dimensional Unsupervised Probabilistic Pose Reconstruction (3D-UPPER) for Freely Moving Animals

Aghileh Ebrahimi ^1^, Patrycja Orlowska-Feuer ^1^, Qian Huang ^1^, Antonio Zippo ^2^, Rasmus S. Petersen ^1^, Riccardo Storchi ^1^

^1^  University of Manchester, Manchester, UK
^2^  Consiglio Nazionale delle Ricerche, Rome, Italy

**Abstract:** A key step in understanding animal behavior relies on the ability to quantify pose and movements. Methods to track body landmarks in 2D have made great progress over the last few years; however, accurate 3D reconstruction of freely moving animals still represents a challenge and suffers from missing values and outliers. To address this challenge here, we develop the 3D-UPPER algorithm, which is fully automated and data-driven, requires no a priori knowledge of the properties of the body and can also be applied to 2D data (2D-UPPER). We show that 3D-UPPER reduces by ~17-fold errors in 3D reconstruction of mouse body during freely moving behavior. To achieve that, 3D-UPPER estimates an Unsupervised Statistical Shape Model (USSM) to constrain viable 3D coordinates. We show, by using simulated data, that our newly developed USSM estimator is robust even in datasets containing up to 50% of poses with outliers and/or missing data. In simulated and real data, USSM estimation converges rapidly, capturing the main directions of change in body shape. Both in 3D and 2D data, such changes were represented by body elongation along the rostro-caudal axis and left/right body torsions, two important quantifier of mouse behaviors. Then, we addressed this question by developing robust 3D video reconstruction of mouse head and body during spontaneous exploration, paired with simultaneous neuronal recordings from dLGN in visual (dark/light) tasks. Our results demonstrate that the primary visual thalamus, beyond global modulations by sleep/awake states, is potentially involved in specific visuomotor integration, and reveal two distinct couplings between up/down postures and neuronal activity. 

### 2.52. Look-Up and Look-Down Neurons in the Mouse Visual Thalamus during Freely Moving Exploration

Patrycja Orlowska-Feuer ^1^, Aghileh Ebrahimi ^2^, Antonio Zippo ^3^, Rasmus S. Petersen ^2^, Robert Lucas ^2^ and Riccardo Storchi ^2^

^1^  Division of Neuroscience and Experimental Psychology, Faculty of Biology,
  Medicine and Health, University of Manchester, Manchester, M13 9PT, UK
^2^  University of Manchester, Manchester, UK
^3^  Consiglio Nazionale delle Ricerche, Rome, Italy

**Abstract:** Visual information reaches the cortex via the thalamic dorsal lateral geniculate nucleus (dLGN). dLGN activity is modulated by global sleep/wake states and arousal, indicating that it is not simply a passive relay station. However, its potential for more specific visuomotor integration is largely unexplored. We addressed this question by developing robust 3D video reconstruction of mouse head and body during spontaneous exploration, paired with simultaneous neuronal recordings from dLGN. Unbiased evaluation of a wide range of posture and movements revealed a widespread coupling between neuronal activity and few behavioral parameters. In particular, postures associated with the animal looking up/down correlated with activity in >50% neurons and the extent of this effect was comparable to that induced by full body movements (typically locomotion). By contrast, thalamic activity was minimally correlated with other postures or movements (e.g., left/right head and body torsions). Importantly, up/down postures and full body movements were largely independent and jointly coupled to neuronal activity. Thus, while most units were excited during full body movements, some expressed highest firing when the animal was looking up (‘look-up’ neurons) while others when the animal was looking down (‘look-down’ neurons). These results were observed in the dark, thus representing a genuine behavioral modulation, and were amplified in a lit arena. Our results demonstrate that the primary visual thalamus, beyond global modulations by sleep/awake states, is potentially involved in specific visuomotor integration, and reveal two distinct couplings between up/down postures and neuronal activity. 

### 2.53. EnLIGHTen the Depressed Brain: Functional and Structural Neural Correlates before and after Bright-Light Therapy in Depression

Oana Georgiana Rus-Oswald ^1^, Emma Visser ^2^, Claudia Simons ^3^, Priscilla Oomen ^4^, Marijke Gordijn ^5,6^, Machteld Marcelis ^7^, Yvonne de Kort ^8^, Luc Schlangen ^8^, Johanna H. Meijer ^9^, Willem van der Does ^10^ and Niki Antypa ^10^

^1^  Department of Clinical Psychology, Leiden University, Leiden, The Netherlands
^2^  Human Technology Interaction group, department of Industrial Engineering and Innovation Sciences, Eindhoven University of Technology, Eindhoven, The Netherlands
^3^  GGzE Mental health institute of Eindhoven and the Kempen, Eindhoven, the Netherlands & Department of Psychiatry and Neuropsychology, School for Mental Health and Neuroscience, Maastricht University, Maastricht, The Netherlands
^4^  GGzE Mental health institute of Eindhoven and the Kempen, Eindhoven & Department of Biomedical Sciences of Cells and Systems and Department of Psychiatry, University of Groningen, University Medical Center Groningen, Groningen, The Netherlands
^5^  Chrono@Work B.V., Groningen, The Netherlands
^6^  Chronobiology Unit, Groningen Institute for Evolutionary Life Sciences, University of Groningen, Groningen, The Netherlands
^7^  GGzE Mental health institute of Eindhoven and the Kempen, Eindhoven, the Netherlands & Department of Psychiatry and Neuropsychology, School for Mental Health and Neuroscience, Maastricht University, Maastricht, The Netherlands
^8^  Eindhoven University of Technology, Industrial Engineering and Innovation Sciences, Eindhoven, The Netherlands
^9^  Department of Neurophysiology, Leiden University Medical Center, Leiden, The Netherlands
^10^  Department of Clinical Psychology, Leiden University, Leiden, The Netherlands

**Abstract: Background**: Bright-Light Therapy (BLT) has been initially used for the treatment of seasonal affective disorder. However, it has also been shown that it can be an effective treatment for other types of depression (e.g., unipolar and bipolar depression). Its positive treatment response being most probably mediated by its efficiency in regulating the disturbed circadian rhythms encountered in these patient populations. Although there are various animal studies evaluating the effect of light on the brain, the exact brain mechanisms underlying BLT responses have not been studied in humans so far. Hence, the aim of this study is to evaluate the effects of BLT and direct light exposure on the brain function before and after a light therapy intervention. Additionally, we aim to establish a model for the brain circuitry involved in the antidepressant effect of BLT. Methods: Within a multicenter randomized controlled clinical trial (RCT) (see Abstract #29), we want to evaluate the neural correlates of BLT before and after treatment, using neuroimaging techniques, i.e., functional and structural MRI. Twenty-four patients with depression (DP) and 24 age and gender matched healthy controls (HC) will receive BLT consisting of a 30 min exposure to a bright light lamp (10,000 LUX) in the morning on 5 consecutive days for a duration of one to three weeks, depending on symptom improvement. Participants will be randomly assigned to one of the three intervention types: (1) light therapy at home, (2) light therapy in a café setting, (3) light therapy in a café combined with blue light blocking glasses, fitted to the individual sleep schedules of the participants. Besides the evaluation of the effects of the three different treatment strategies on depressive symptoms and sleep, participants will undergo MRI scans at baseline and post-treatment to assess brain markers of treatment response. Both structural as well as functional scans will be performed addressing the whole brain but also specific regions of interest (e.g., suprachiasmatic area, amygdala, habenula). Expected results: We expect a difference in the functional activity and connectivity of light-stimulated areas between DP and HC. Furthermore, we expect that BLT, when successful, reduces these differences and that such reductions can be linked to improvements in symptomatology. Conclusion: The results of this study will contribute to a better understanding of the exact mechanisms which make BLT a successful antidepressant treatment, not only on a chronobiological but also on a neural and behavioral level. Furthermore, the identification of treatment response predictors is of high clinical relevance, as this enables the optimization of chronotherapeutic personalized interventions and the establishment of clinical guidelines to improve well-being and facilitate recovery.

### 2.54. Light Therapy in Neurodegenerative Disorders

Aleksandar Videnovic

Boston, USA

**Abstract:** Light therapy (LT) is a well-established treatment modality in sleep medicine and psychiatry. Activation of the suprachiasmatic nucleus, the pacemaker of the circadian system, and direct alerting effects are major mechanisms by which supplemental light exposure has beneficial effects on mood, sleep and circadian rhythms. Among neurodegenerative disorders, LT has been most used in Alzheimer’s disease (AD). Several investigations identified the benefits of LT on diurnal rest-activity rhythms in AD. Bright LT of 10,000 lux is associated with improved sleep quality and duration. Light exposure as low as 2500 lux during the morning and evening has a beneficial effect on circadian rhythms, altered circadian phase and sleep in individuals with AD residing in nursing homes. Other studies demonstrated improvements in alertness but no considerable improvement in self-reported sleep metrics in AD. A novel dawn–dusk simulation technology is associated with the enhanced effectiveness of external zeitgebers and improvement in mood in individuals with AD. Research in Parkinson’s disease (PD) and LT is at an early stage. Several studies have demonstrated the beneficial effects of LT in patients with PD with limited reported side effects. LT investigations in the PD population reported to date differ in the intensity of LT (300–10,000 lux), duration of LT exposure (30–90 min) and timing of LT (evening, morning or both). These investigations documented improvements in mood and several aspects of sleep and alertness, such as sleep-onset latency, sleep continuity, sleep quality and excessive sleepiness. Bright LT also improved bradykinesia and rigidity, which led to the successful reduction of dopamine-replacement therapy. Overall, a growing body of evidence supports the potential benefit of LT for the improvement of circadian, sleep and neuropsychiatric symptoms of AD and PD. Further work will need to focus on determining the optimal timing, duration, frequency of exposure and the optimal wavelength of light therapy as a treatment modality for neurodegenerative disorders. 

### 2.55. The BioClock Consortium: Translating Fundamental Chronobiology to Practical Applications for a Healthy Circadian Clock

Laura Kervezee

Department of Neurophysiology, Leiden University Medical Center, Leiden, The Netherlands

**Abstract: Introduction**: The timing of light exposure, physical activity and food intake are important cues for synchronizing the circadian timing system. However, these inputs have drastically—and abruptly—changed in our modern society due to the widespread use of artificial light and the round-the-clock demand for goods and services, posing a challenge to circadian clock function in humans and the organisms around us. Although a wealth of fundamental knowledge has been generated in the field of chronobiology over the past decades, the translation of this knowledge to practical applications is limited. **Methods**: Addressing these chronobiological challenges requires a close collaboration between complementary disciplines. To achieve this, we established the BioClock consortium, a Dutch research network of molecular biologists, neuroscientists, ecologists, clinicians and psychologists working closely together with educators, policy makers, local governments, environmental organizations, industry and engaged citizens in order to capitalize on results obtained from fundamental chronobiological research, thereby helping to promote human health, quality of life, and biodiversity. **Results**: Upon receiving funding from the Dutch Research Council (NWO), 25 PhD students and postdocs were recently recruited at institutes across the Netherlands to work towards BioClock’s objectives, focusing on three linked and complementary clusters of research questions related to the biological clock’s role in: (1) society, (2) healthcare and (3) the natural environment. Dedicated outreach activities were designed to foster connections with relevant stakeholders and the general public. **Conclusion**: Operating at a national level, the BioClock consortium is well positioned to combine fundamental research with practical interventions in order to create a sustainable living environment that preserves circadian clock function in humans and the organisms around us. In this presentation, I will provide an overview of the BioClock consortium, highlight the plans for the upcoming years and present some of our preliminary findings. 

### 2.56. Chronobiology Meets Lighting Design: New Light for Air Traffic Controllers

Oliver Stefani ^1^, Inge Sommerlatte ^2^, Lucia Beatriz Palmero ^3^, Jennifer Burkhalter ^4^, Rene Lehner ^4^ and Christian Cajochen ^1^

^1^  University of Basel, Basel, Switzerland
^2^  Sommerlatte & Sommerlatte Lichtplanung, Zürich, Switzerland
^3^  University of Murcia, Murcia, Spain
^4^  Skyguide Air Navigation Service ACC East, Wangen, Switzerland

**Abstract:** Current luminaires at the Swiss air navigation centre (Skyguide) led to reduced visual comfort among air traffic controllers. We measured vertical illuminances at eye level below the current recommendations for healthy daytime lighting conditions. With a new lighting design, we achieved four times higher vertical ‘melanopic equivalent daylight illuminances’ (mEDI) during the day and three times lower mEDI during the night. The results of a survey with the air traffic controllers before and after the installation of the new lighting concept indicated a clear and significant improvement in the perceived naturalness of the new lighting design. Subjective well-being also increased with the new design while the subjective glare ratings did not change. We conclude that good lighting design and chronobiology can complement each other. **Introduction**: How can lighting optimally support the work of air traffic controllers, create good visual conditions, increase concentration and well-being, and strengthen the sleep–wake rhythm? We asked these questions at the beginning of a project to renew and improve the lighting conditions in the control centre at Skyguide in Wangen near Dübendorf, Switzerland. Skyguide, the Swiss air navigation service provider, controls the Swiss airspace as well as some adjacent airspaces in France and Germany. High luminance differences between existing luminaires and the dark-appearing ceiling led to direct glare and thus reduced visual comfort among the air traffic controllers. Thus, the new lighting concept required a balanced luminance distribution in the room. **Methods**: Analysis of the existing lighting situation at workplaces revealed vertical melanopic EDIs [1] at a height of 1.2 m in the range of 28 lx to 55 lx, which is clearly below the current recommendations of 250 lx mEDI for healthy daytime lighting conditions [2]. Working at computer screens, however, offers only limited possibilities of lighting applications. For example, luminance differences must not exceed threshold values (e.g., luminance ratio between the working field and its immediate surroundings <3:1) and glare must be avoided. For the new lighting concept, we sought quantification of light in terms of its spectral distribution, temporal and spatial dynamics and illuminance levels at the eye in order to achieve a physiological impact on humans in terms of concentration capacity, well-being but also strengthening sleep–wake rhythms. Ensuring the safe transport of people around the clock means an immense responsibility for air traffic controllers. Thus, an important aspect of the new lighting design was implementing the role of the non-image-forming photoreceptors (‘ipRGCs’) (3) located in the retina and their influence on human sleep–wake rhythms, concentration and sleepiness/fatigue [4,5]. These effects are often described as a non-visual light effect. Unfortunately, compliance with current lighting standards, which only consider the visual aspects of light, usually results in little non-visually effective light reaching the eye. The new lighting concept at Skyguide was guided by a recently published practice report, ‘Should We Re-think Regulations and Standards for Lighting at Workplaces? A Practice Review on Existing Lighting Recommendations’ [6]. In this review, existing regulations and standards on visual lighting aspects are contrasted with new recommendations on non-visual aspects and highlight conflicts between them. Here we aimed at combining current guidelines for lighting to avoid glare with new lighting recommendations for non-visual effects in workplaces through the following ideas:

1. Selecting an appropriate spectrum by using light sources with a relatively high mEDI [1] since the non-visual effects are mainly related to mEDIs [7,8] and not necessarily to the color temperature or illuminance.
2. Enhancing the vertical illuminance at the eye by optimizing the light distribution (also taking into account the reflection of the surrounding surfaces). An optimized lighting design should achieve higher vertical illuminances at the eye with the same horizontal illuminance. Often, the downward lighting of ceiling luminaires intended for rooms with PC monitors results in relatively low vertical illuminance levels. To achieve higher vertical illuminances and thus more light at the eye, we implemented floor-mounted luminaires with indirect light distribution (directing light to the ceiling).
3. Since the iPRGCs are distributed over a large area of the retina [3], it can be assumed that non-visual effects are greatest when the light comes from a large-area source. If only a small area of the retina is illuminated, as is the case with the directional light from a spotlight, a weaker non-visual effect is assumed. For this reason, in addition to the spectral change, the light distribution between day and night was changed in such a way that during daytime the large ceiling is illuminated, whereas during nighttime suspended luminaires illuminate local work areas.

**Results**: Designing an optimal, dynamic light pattern (light spectrum, light distribution and illuminance) to promote well-being and performance was particularly challenging for shift-working air controllers. We successfully achieved this objective through predominantly indirect lighting. Asymmetrically radiating ceiling lights integrated into the wall design and light poles in the centre of the room complement each other to illuminate the large ceiling evenly. The luminaires were equipped with LEDs with a color temperature of 5700 K and a color rendering CRI > 92. The air traffic controllers’ workstations were additionally supplied with a suspended luminaire. The color temperature and luminous flux of all workstation luminaires can be controlled by a central lighting control system. With the new lighting solution, it is possible to change light characteristics across day and night. During normal flight operations at daytime, indirect lighting provides glare-free background lighting. The suspended luminaires emit a color temperature of 5700 K, coherent with the indirect lighting. During nighttime, with far less air traffic from 23:30 to 6:00, lighting is very much reduced. Only the pendant lights above the supervisors’ area emit light with a color temperature of 2700 K and at a very low illuminance (22–28 lx vertical at the eye). They provide a small island of light above an individual work area at night. This is based on the fact that light at night should minimally affect biological rhythms but still be of good quality for the visual system. Photographs of the new lighting concept are depicted in Figure 1 and Figure 2. With the new lighting concept, we achieved a vertical mEDI of 110–263 lx at a height of 1.2 m during the day, dependent on the measurement location. During night shifts, these values decreased to 13–17 lx mEDI. It has to be noted that during night shifts, there are far fewer flights and the workload is lower than during the day. We conducted a survey on subjective well-being and perceived light quality with the air traffic controllers (n = 110). This occurred first in September 2020 in the original lighting condition and in September 2021 after installing the new lighting concept, which took place in February 2021. The results indicate a significant improvement in the perceived naturalness of light (*p* < 0.01, see Figure 3C) and subjective well-being (*p* = 0.032 and *p* = 0.019, see Figure 3A,B) after implementing the new lighting concept. The subjective perception of glare did not significantly change (*p* = 0.478) between the original and new lighting solution. **Conclusions**: Integrating recommendations for healthy daytime lighting conditions with good visual conditions for computer work can be achieved by a balanced luminance distribution in the room. Besides a spectral change, the biological rhythm and psychological well-being of shift workers can be improved by additionally changing the light distribution between day and night and thus the incident light at the eye.

**References**

1. 026/E:2018 CS. CIE System for Metrology of Optical Radiation for ipRGCInfluenced Responses to Light. Color Research & Application. 2018;44(2):316-.
2. Brown T BG, Cajochen C, Czeisler C, Hanifin J, Lockley S, Lucas R, Munch M, O’Hagan J, Peirson S, Price L, Roenneberg T, Schlangen L, Skene D, Spitschan M, Vetter C, Zee P, Wright Jr K. Recommendations for Healthy Daytime, Evening, and Night-Time Indoor Light Exposure. Preprints. 2020.
3. Hattar S, Liao HW, Takao M, Berson DM, Yau KW. Melanopsin-containing retinal ganglion cells: architecture, projections, and intrinsic photosensitivity. Science. 2002;295(5557):1065-70.
4. Souman JL, Tinga AM, Te Pas SF, van Ee R, Vlaskamp BNS. Acute alerting effects of light: A systematic literature review. Behav Brain Res. 2018;337:228-39.
5. Cajochen C, Munch M, Kobialka S, Krauchi K, Steiner R, Oelhafen P, et al. High sensitivity of human melatonin, alertness, thermoregulation, and heart rate to short wavelength light. J Clin Endocrinol Metab. 2005;90(3):1311-6.
6. Stefani O, Cajochen C. Should We Re-think Regulations and Standards for Lighting at Workplaces? A Practice Review on Existing Lighting Recommendations. Frontiers in Psychiatry. 2021;12.
7. Brown TM. Melanopic illuminance defines the magnitude of human circadian light responses under a wide range of conditions. Journal of Pineal Research. 2020;69(1):e12655.
8. Giménez M, Stefani O, Cajochen C, Lang D, Deuring G, Schlangen LJM. Predicting melatonin suppression by light in humans: unifying photoreceptor-based equivalent daylight illuminances, spectral composition, timing and duration of light exposure. Journal of Pineal Research.n/a(n/a):e12786.

### 2.57. Neuroimaging the Impact of Light on the Interactions between Subcortical Areas and the Cortex

Gilles Vandewalle

Sleep & Chronobiology Laboratory, GIGA-Cyclotron Research Centre-In Vivo Imaging-ULiège-Belgium

Exposure to blue light stimulates alertness and cognitive performance in humans. Neuroimaging studies established that this stimulating effect of light affects a wide spread of brain regions depending on the ongoing cognitive process. Results of these studies were compatible with a scenario in which light information would affect first subcortical regions involved in attention and alertness regulation prior to modulating non-visual regional cortical activity. Our latest research aims to provide empirical demonstration of this putative scenario in humans. Healthy young individuals were exposed to cold (‘blueish’) polychromatic light (4000 K) of different irradiance and a control monochromatic orange light (590nm; 5.3 × 1012 photons/cm^2^/s) while performing cognitive tasks in an Ultra-High Field 7 Tesla MRI scanner. Analyses suggest that different parts of the hypothalamus are differentially affected by the blueish light exposure. In addition, analyses further reinforce the idea that activity of the pulvinar and other posterior nuclei of the thalamus are consistently modulated by light exposures during a non-visual cognitive task in humans. Connectivity analyses further showed that blueish light, but not orange light, specifically modulated pulvinar-to-parietal cortex connection. These latest results provide empirical evidence suggesting that blue light affects cognitive activity by modulating information flow from subcortical to cortical areas. 

## Figures and Tables

**Figure 1:**
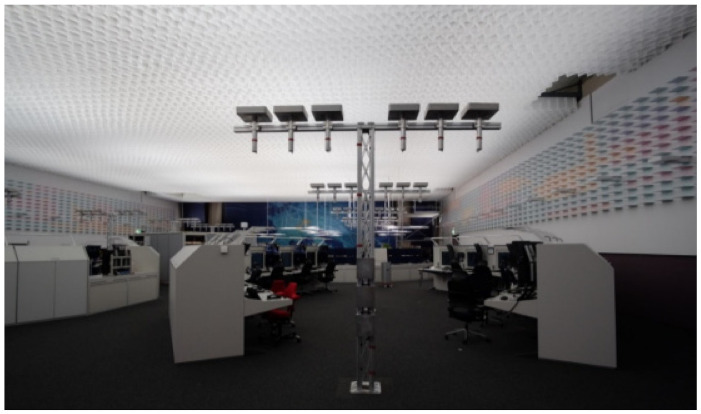
Light scenario during the day when workload is high.

**Figure 2:**
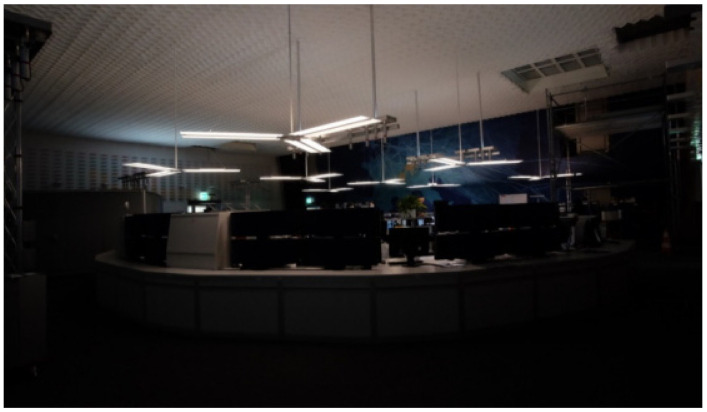
Light scenario during the night when workload is low A B C.

**Figure 3:**
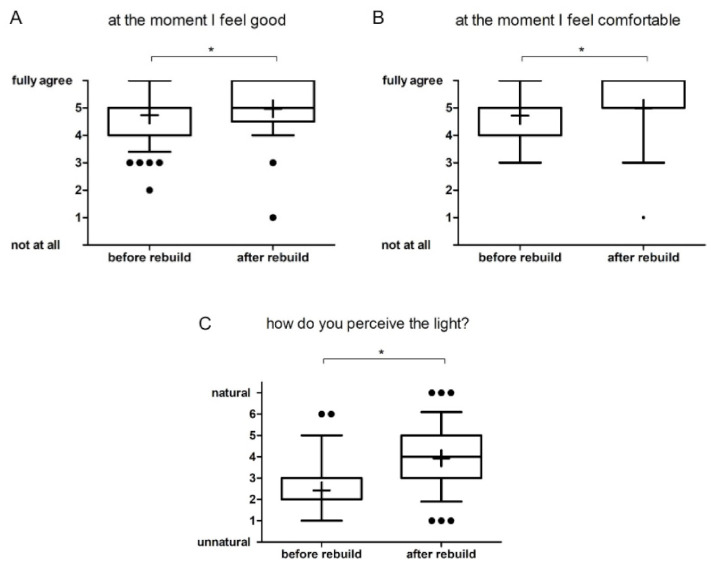
(**A**–**C**): Results of the survey.

